# New Gold(I) Complexes as Potential Precursors for Gas-Assisted Methods: Structure, Volatility, Thermal Stability, and Electron Sensitivity

**DOI:** 10.3390/molecules30010146

**Published:** 2025-01-02

**Authors:** Aleksandra Butrymowicz-Kubiak, Tadeusz M. Muzioł, Piotr Madajski, Iwona B. Szymańska

**Affiliations:** Faculty of Chemistry, Nicolaus Copernicus University in Toruń, Gagarina 7, 87-100 Toruń, Poland; aleksandra.butrymowicz@doktorant.umk.pl (A.B.-K.); piotr.madajski@doktorant.umk.pl (P.M.)

**Keywords:** chemical vapor deposition, focused electron beam-induced deposition, focused ion beam-induced deposition, Hirshfeld surface analysis, volatile compounds, volatility study, nanomaterials, electron interactions, gold(I) complexes, thermal properties

## Abstract

We report the synthesis and characterization of new, user-friendly gold(I) [Au_4_(μ-(NH)_2_CC_2_F_5_)_4_]_n_ coordination polymer and [Au_2_Cl_2_(NH_2_(NH=)CC_2_F_5_)_2_]_n_ complex. These compounds were investigated for potential application as precursors in chemical vapor deposition (CVD) and focused electron/ion beam-induced deposition (FEBID/FIBID), which are additive methods to produce nanomaterials. Single-crystal X-ray diffraction, elemental analysis, and infrared spectroscopy were used to determine the complexes’ composition and structure. We studied their thermal stability and volatility using thermal analysis and variable-temperature infrared spectroscopy (VT IR) and by conducting sublimation experiments. The gold(I) amidinate [Au_2_(μ-(NH)_2_CC_2_F_5_)_2_]_n_ sublimates at 413 K under 10^−2^ mbar pressure. The electron-induced decomposition of the complexes’ molecules in the gas phase and of their thin layers on silicon substrates was analyzed using electron impact mass spectrometry (EI MS) and microscopy studies (SEM/EDX), respectively, to provide insights for FEBID and FIBID precursor design. The [Au_2_Cl_2_(NH_2_(NH=)CC_2_F_5_)_2_]_n_ hydrogen chloride molecules evolved during heating, with the formation of gold(I) amidinate. The obtained results revealed that the new gold(I) amidinate may be a promising source of metal for nanomaterial fabrication by gas-assisted methods.

## 1. Introduction

Gold is an ideal candidate for modern industrial applications due to its high conductivity and resistance to electromigration, which makes it valued for use in electrical contacts [1,2]. Furthermore, this metal has attracted increasing attention for its plasmonic and optoelectronic applications, e.g., in direct nanoemitters or high-performance sensors, integrated optical circuits, and optoelectronic devices [3,4,5]. Gold nanoparticles with a homogenous size distribution are used in Surface-Enhanced Raman Spectroscopy (SERS), an important field of plasmonic research. Moreover, they are invaluable in new anti-cancer therapies, where they are used for targeted drug delivery or bioimaging [6,7,8,9,10].

The ability of miniaturization is an essential aspect of modern technology. Fabrication at the nanoscale level often involves the formation of 2D and 3D nanomaterials with different functionalities. Nanodeposits with outstanding magnetic, electrical, optical, mechanical, and catalytic properties can be produced by various gas-assisted methods, e.g., chemical vapor deposition (CVD), atomic layer deposition (ALD), focus electron beam-induced deposition (FEBID), and focus ion beam-induced deposition (FIBID) [11,12,13,14]. These processes need coordination compounds as the metal source in the gas phase [6,11]. CVD is a widely used method in which deposits are formed on a heated substrate by a decomposition reaction of adsorbed precursor molecules [15]. ALD differs from CVD in the introduction of a gas precursor. In ALD, two compounds are introduced separately in pulses and undergo sequential and self-limiting surface reactions [16]. FEBID and FIBID are relatively new emerging methods. These direct deposition techniques for nanofabrication also need volatile and appropriately sensitive precursors. Molecules are adsorbed on a substrate surface and decomposed under the influence of a focused electron or ion beam (keV) [6,11]. In the FEBID process, the influence of secondary electrons (eV) produced from the substrate is essential for nanostructure growth. This type of electrons are also significant in the FIBID process, where they are generated by a focused ion beam. Therefore, combining volatility with electron/ion sensitivity and thermal stability is crucial for designing new FEBID and FIBID precursors.

Commercially available CVD precursors are often tested in FEBID and FIBID processes. However, the applied gold compounds are air-sensitive and thermally unstable, which makes their storage and fabrication problematic for FEBID and FIBID processes. Therefore, new and user-friendly gold precursor molecules are needed to overcome this issue. High-purity gold films have been generated by the CVD method from a number of volatile gold complexes. Some of the most important gold compounds used are β-diketonates and complexes with the so-called small ligands [Au_2_Me_4_(µ-Cl_2_)], [AuCl(SMe_2_)], [AuCl(PMe_3_)], and [AuMe(PMe_3_)] [6,17]. In contrast, the amount of FEBID and FIBID precursors is limited. The first FEBID experiments on gold(III) β-diketonate complexes were carried out using [Au(acac)Me_2_] (acac–acetylacetonate), [Au(tfac)Me_2_] (tfac–trifluoroacetylacetonate), and [Au(hfac)Me_2_] (hfac–heksafluoroacetylacetonate). The metal content in the deposits varied in the range of 30–39 at.% Au. The highest value, 91 at.% Au, was achieved for [Au(tfac)Me_2_] in FEBID experiments when co-injected water was applied as the oxidizing gas, while the gold(I) complexes [AuCl(PF_3_)] and [AuCl(CO)] produced high-purity FEBID deposits at the level of 95–100 at.% Au without post-deposition purification. However, these precursors are highly air-sensitive, which complicates their practical use [3,6,18]. In the case of the FIBID method, only [Au(hfac)Me_2_] has been tested (75 at.% Au) [11,19,20].

One group of volatile coordination compounds is that of non-fluorinated amidinates such as [Au((N^i^Pr)_2_CMe)_2_], [Au((N^i^Pr)_2_C^n^Bu)_2_] [21], and gold(I) 5,5-bicyclic amidinate [22], studied for their potential application as precursors in atomic layer deposition (ALD) and chemical vapor deposition (CVD). However, these compounds have not been even preliminary studied for their applicability in electron- or ion-driven processes. Relatively new compounds are perfluorinated amidinates with the general formula (NH)_2_CR_f_–(R_f_—perfluorinated group). On the other hand, perfluorinated substituents increase compound volatility due to reduced interactions between molecules caused by the repulsion of the fluorine atoms [23]. Among the perfluorinated compounds, are silver(I) and mercury(II) complexes such as [Ag((NH)_2_CCF_3_)], [Ag((NH)_2_CC_2_F_5_)], [Hg((NH)_2_CC_2_F_5_)_2_] [24], which have not been investigated for use in gas phase deposition processes. Only the mentioned silver(I) and new copper(I) amidinates have been recently studied by us for their potential application in the FEBID and FIBID processes by electron interaction investigations [25].

Based on that motivation, we here present a synthesis method for new air-stable gold(I) [Au_4_(µ-AMDC_2_F_5_)_4_]_n_ (AMD–(NH)_2_CC_2_F_5_) and [Au_2_Cl_2_(HAMDC_2_F_5_)_2_]_n_ (AMDH–NH_2_(NH=)CC_2_F_5_) complexes containing the perfluorinated C_2_F_5_ substituent that should be promising for their volatility features. Furthermore, we demonstrate the crystal structure and intermolecular interactions of the molecules based on Hirshfeld surface analysis for a gold(I) amidinate. This approach can be employed to prescreen and score new potential precursors for vapor deposition methods. The compounds’ thermal stability was determined under atmospheric pressure, and their suitability for the production of metallated volatile species under reduced pressure was studied. Moreover, the low-energy electron interactions of Au(I) amidinate in the gas phase was investigated. The electron beam sensitivity of gold(I) amidinate was studied using electron microscopy imaging. Furthermore, a thermal decomposition mechanism for [Au_2_Cl_2_(HAMDC_2_F_5_)_2_]_n_ is proposed. Therefore, we investigated whether new user-friendly complexes can be used in CVD and as an alternative to the unstable gold(I) and gold(III) compounds previously tested in FEBID and FIBID [6].

## 2. Experimental Section

### 2.1. Materials

Anhydrous acetonitrile (99.8%), chloro(dimethylsulfide)gold(I) [Au(SMe_2_)Cl] (>97.0%), and tetrahydrothiophene (tht) (99%) were purchased from Sigma Aldrich (Saint Louis, MO, USA), HAuCl_4_∙xH_2_O (≥99.9%) from Pol-Aura (Zawroty, Poland), AgNO_3_ (99.9%) from Chempur (Piekary Śląskie, Poland), C_2_F_5_C(=NH)NH_2_ (HAMD–C_2_F_5_) (98.7%) from Apollo Scientific (Stockport, UK), and ethanol (96%) and NaHCO_3_ (p.a) from Avantor Performance Materials Poland (Gliwice, Poland). [Au(tht)Cl] [26] and [Ag_2_((NH)_2_CC_2_F_5_)_2_] [25] were prepared as earlier reported. Ag_2_CO_3_ was synthesized in the reaction of AgNO_3_ and NaHCO_3_, as described previously [27]. All reagents were used as received. The Si(111) substrates were purchased from the Institute of Microelectronics and Photonics, Center for Electronic Materials Technology in Warsaw–Lukasiewicz Research Network (Warsaw, Poland).

### 2.2. Instrumentation

IR spectra were registered with a Vertex 70V spectrometer (Bruker Optik, Leipzig, Germany) using a reflective single-reflection diamond ATR unit (200–4000 cm^−1^). Electron impact mass spectra (EI MS) were registered using an AutoSpec Premier, Waters Corporation (Milford, MA, USA) over the temperature range of 313–573 K. The C, H, and N content was determined using a Vario MACRO CHN ELEMENTAR Analysensysteme (GmbH, Langenselbold, Germany). Thermal studies (TGA/DTA) were performed using an SDT 2960 TA analyzer (New Castle, DE, USA; dry N_2_; heating rate 2.5 K min^−1^), in a heating range up to 1273 K and a sample mass of 3 and 10 mg. Variable-temperature infrared spectra (VT IR) were registered using a PerkinElmer Spectrum 2000 spectrometer (Waltham, MA, USA) over the range of 400–4000 cm^−1^ with a medium slit width and a peak resolution of 2.0 cm^−1^. A glass vessel with the precursor sample (~100 mg) was placed in a homemade reactor tube and heated (from 333 to 693 K) under a dynamic vacuum (p = 10^−1^ mbar). The sublimation experiments were performed under reduced pressure (10^−2^ mbar) in a glass sublimator with a special holder for a silicon substrate. The morphology and composition studies of layers of the compounds were performed using scanning electron microscopy with Quanta 3D FEG, FEI (Hillsboro, OR, USA) and SEM–LEO 1430VP, Ltd., (Cambridge, UK) microscopes (operating voltage 8 and 20 kV), equipped with the energy-dispersive X-ray spectrometer (EDS) Quantax 200 with an Xflash 4010 detector Bruker AXS microanalysis GmbH (Berlin, Germany), which was also used in preliminary electron sensitivity tests. Transmission electron microscopy, TEM G2 F20X-Twin 200 kV FEI (Hillsboro, OR, USA) was used to characterize the thermal analysis residue and test the sensitivity of the compounds to high-energy electrons (200 keV). The samples for the TEM experiments were prepared by dissolving the compounds in anhydrous ethanol (99.8%), applying a drop on a carbon-coated copper mesh with holes (Lacey type, 400 mesh), and evaporating the solvent at room temperature. Energy-dispersive X-ray spectroscopy, EDX, RTEM model SN9577, 134 eV, EDAX, FEI, (Hillsboro, OR, USA) spectra and selected-area (electron) diffraction patterns were recorded to identify the chemical compositions.

### 2.3. Software

All graphical data were further processed with OriginPro version 9.1. TA Universal Analysis (New Castle, DE, USA) was used to analyze the thermograms. To assign the decomposition products in VT IR experiments, the registered spectra were compared with the spectra of molecules in the gas phase collected in SpectraBase (https://spectrabase.com/, accessed on 12 July 2024) [28]. Diagrams indicating the synthesis of the compounds (**1**), (**2**) and the VT IR mechanism were drawn using ChemDraw Ultra version 12.0 (Cambridge, MA, USA). CrystalExplorer 21.5 (revision 608bb32) [29] was used to calculate Hirshfeld surfaces. All figures of the crystal structures were prepared using DIAMOND 4 version 4.6.8 [30] and ORTEP-3 (version 1.0.3) [31] software.

### 2.4. Synthesis

#### 2.4.1. Synthesis of [Au_4_(μ-(NH)_2_CC_2_F_5_)_4_]_n_ (Denoted, for Convenience, [Au_4_(µ-AMDC_2_F_5_)_4_]_n_) (**1**)

[Au(SMe_2_)Cl] (0.28 mmol) was added to 20 cm^3^ of anhydrous acetonitrile; then, [Ag_2_((NH)_2_CC_2_F_5_)_2_] (0.14 mmol) was introduced to the solution, which was stirred for 24 h. The silver(I) chloride formed during the reaction was filtered and washed with acetonitrile. The solvent from the filtrate was removed under reduced pressure (10^−2^ mbar) to obtain a white solid, insoluble in available organic solvents and stable in argon atmosphere and air for months. Yield: 73% (**1**). White single crystals suitable for X-ray structure analysis were grown by slow evaporation from the mother liquor for complex (**1**) at room temperature.

#### 2.4.2. Synthesis of [Au_2_Cl_2_(NH_2_(NH=)CC_2_F_5_)_2_]_n_ (Denoted, for Convenience, [Au_2_Cl_2_(HAMDC_2_F_5_)_2_]_n_) (**2**)

[Au(tht)Cl] (0.12 mmol) was added to 20 cm^3^ of anhydrous acetonitrile; then, C_2_F_5_CNHNH_2_ (0.12 mmol) was introduced to the solution and stirred for 24 h. The mixture was then filtered, and the solvent from the filtrate was removed under reduced pressure (10^−2^ mbar) to obtain a white-pink solid, insoluble in available organic solvents and stable in argon atmosphere for months and in air for weeks. Yield: 43% (**2**). No single crystals suitable for X-ray structure analysis were obtained for compound (**2**), despite efforts to grow them.

**[Au_4_(µ-AMDC_2_F_5_)_4_]_n_** (**1**) Au_4_N_8_H_8_C_12_F_20_ (calc./found) % H 0.56/0.47, % C 10.06/9.84, % N 7.83/7.87; EI MS T = 328 K (*m*/*z*, RI %) [Au(NHCNH)]^+^ (239, 4), [Au(NHNH_2_CCF)]^+•^ (271, 1), [Au_2_(NHNHCC_2_F_5_)_2_]^2+^/[Au(NHNHCC_2_F_5_)]^+•^ (358, 2), [Au_2_(NH_2_)]^+^ (410, 1), [Au_2_(NHCNH)]^2+^ (436, 3), [Au_2_(NHNHCC_2_F_5_)]^+^ (555, 21), [Au_2_(NHNHCC_2_F_5_)(HCN)]^+^ (582, 6), [Au_2_(NHNHCC_2_F_5_)(NHCNH)]^+^ (597, 19), [Au_2_(NHNHCC_2_F_5_)_2_]^+•^ (716, 100), [Au_3_(NHNHCC_2_F_5_)_2_]^+^ (913, 2), [Au_4_(NHNHCC_2_F_5_)]^+^ (949, 8); IR (3412 (m), 3344 (m), 1614 (s), 1512 (w), 1325 (m), 1202 (s), 1171 (s), 1146 (s), 1030 (s), 826 (w), 748 (m), 685 (m), 563 (m), 455 (w), 365 (w), 311 (w) cm^−1^). Yield: 73%.

**[Au_2_Cl_2_(HAMDC_2_F_5_)_2_]_n_** (**2**) Au_2_N_4_H_6_C_6_F_10_Cl_2_ (calc./found) % H 0.77/0.90, % C 9.13/9.81, % N 7.10/7.23; EI MS T = 339 K (*m*/*z*, RI %) [HCl]^+•^ (36, 17), [H_2_^35^Cl]^+^ (37, 67), [H_2_^37^Cl]^+^ (39, 22), [Au(NH)]^+•^ (212, 11), [Au(HNCNH_2_)]^+^ (240, 3), [Au(NHNH_2_CCF)]^+•^ (271, 2), [Au(NHC_2_F_5_)]^+•^ (331, 2), [Au(NNCC_2_F_5_)]^+•^ (356, 5), [Au_2_(NHNHCC_2_F_5_)_2_]^2+^/[Au(NHNHCC_2_F_5_)]^+•^ (358, 6), [Au_2_(NHNHCC_2_F_5_)_2_]^+•^ (716, 4), [Au_3_(NHNCC_2_F_5_)]^+^ (751, 3); IR (3499 (w), 3360 (w), 3233 (m), 3163 (m), 1678 (s), 1611 (m), 1491 (w), 1429 (w), 1387 (w), 1329 (m), 1286 (w), 1213 (s), 1186 (s), 1155 (s), 1024 (s), 804 (w), 775 (w), 735 (w), 694 (w), 625 (m), 600 (m), 565 (w), 527 (w), 484 (w), 434 (w), 386 (m), 338 (m), 247 (w) cm^−1^). Yield: 43%.

### 2.5. X-Ray Crystal Structure Determinations

Single-crystal diffraction data for compound (**1**) were first collected at MX14-2 beamline (BESSY II synchrotron (HZB, Berlin, Germany) at 100 K. The structure was processed using *xdsapp* [32,33]. The structure was solved using direct methods and refined with a full-matrix least-squares procedure on F^2^ (SHELX-2018/1) [34]. All heavy atoms were refined with anisotropic displacement parameters. Hydrogen atoms of NH groups were found from the difference electron density map and refined with isotropic thermal displacement parameters fixed at a value 20% higher than that of the corresponding nitrogen atoms. However, due to the gold presence resulting in a high absorption coefficient, also high difference peaks were observed. Hence, the data collection was repeated at 100 K using a XtaLAB Synergy (Dualflex) diffractometer (Rigaku) with a HyPix detector, MoKα radiation λ = 0.71073 Å. Data reduction was performed in CrysAlis Pro for the studied compound [35], and the Gaussian absorption correction was applied. Those data were used for the final refinement steps with the model from the synchrotron data. The data collection and refinement results are summarized in Supplementary Materials (Table S1), such as selected bond lengths and angles (Table S2). CCDC 2378410 contains supplementary crystallographic data for compound (**1**). These data can be freely obtained from The Cambridge Crystallographic Data Centre via www.ccdc.cam.ac.uk.data_request/cif.

## 3. Results and Discussion

**Figure 1:**
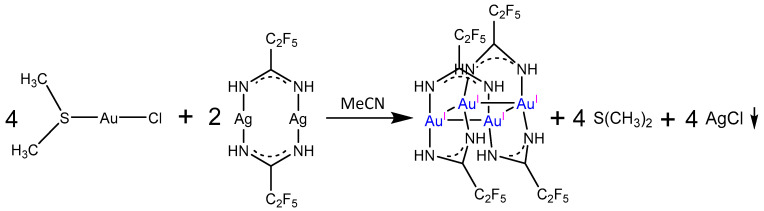
Reaction scheme for the synthesis of the gold(I) complex [Au_4_(µ-AMDC_2_F_5_)_4_]_n_ (**1**).

In anhydrous acetonitrile, chloro(dimethylsulfide)gold(I) reacts with silver(I) amidinate to produce gold(I) amidinate (**1**). The reaction proceeds with the precipitation of silver(I) chloride (Figure 1).

**Figure 2:**
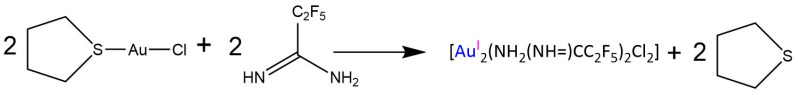
Reaction scheme for the synthesis of the gold(I) complex [Au_2_Cl_2_(HAMDC_2_F_5_)_2_]_n_ (**2**).

Chloro(amidine)gold(I) (**2**) was formed in the reaction of chloro(tetrahydrothiophene)gold(I) with amidine in anhydrous acetonitrile (Figure 2).

The obtained compounds are white (**1**) or white-pink solids (**2**), both being stable for months in argon atmosphere, whereas in air, (**1**) is stable for months, and (**2**) for weeks.

### 3.1. Crystallography

**Figure 3:**
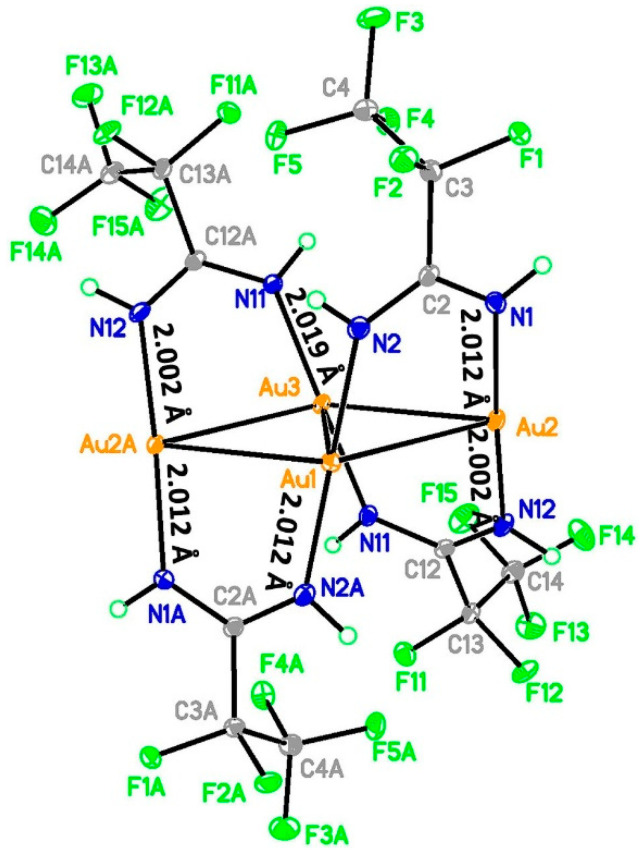
The tetramer structure of [Au_4_(µ-AMDC_2_F_5_)_4_]_n_ (**1**) with ellipsoids at the level of 30% probability (yellow—gold atoms; blue—nitrogen atoms; gray—carbon atoms; green—fluorine atoms).

**Figure 4:**
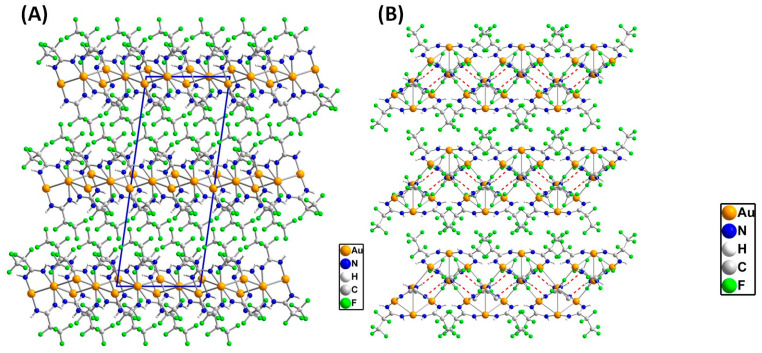
The crystal network of [Au_4_(µ-AMDC_2_F_5_)_4_]_n_ (**1**) along the *b* axis (in blue is marked the unit cell) (**A**) and the crystal structure projected in the *ab* plane (the hydrogen bonds are marked with a red dashed line) (**B**) (yellow—gold atoms; blue—nitrogen atoms; gray—carbon atoms; green—fluorine atoms).

**Figure 5:**
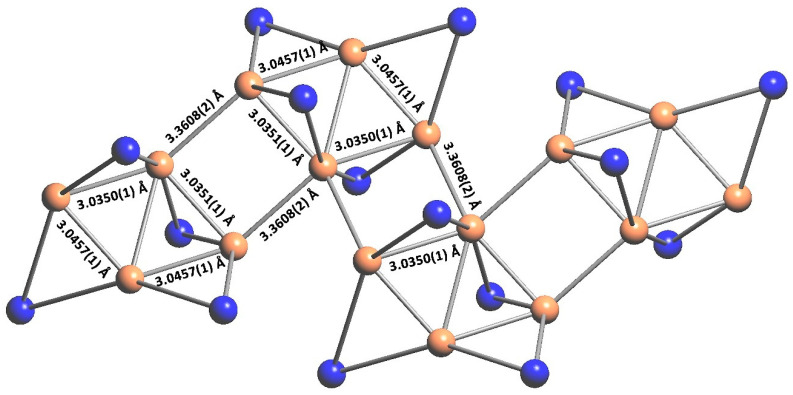
The chain topology in [Au_4_(µ-AMDC_2_F_5_)_4_]_n_ (**1**) shows the zigzag-pattern axis (gold in orange, AMD in blue) based on the analysis performed in TOPOS (Version 5.5.2.2) [38].

[Au_4_(µ-AMDC_2_F_5_)_4_]_n_ (**1**) is a coordination polymer and crystallizes in the monoclinic *I*2/*a* space group with half of the molecule shown by the above formula in the asymmetric unit and Au1 and Au3 atoms located at the twofold axis (Figure 3, Figure 4 and Figure S1, Tables S1 and S2). A tetramer, composed of four gold(I) cations forming an almost perfect rhombus (the Au…Au…Au angles are 66.38 and 113.92°) is the principal motif. The gold(I) cations are connected by amidinate anions as well as aurophilic interactions, with Au…Au distances being 3.035 and 3.046 Å. The [Au_4_(µ-AMDC_2_F_5_)_4_] tetramers are connected by aurophilic interactions into zigzag chains running along the *a* axis, with Au1…Au2 distances being 3.361 Å (only slightly longer than 3.329 Å—the Au1…Au3 separation in the tetramer) and the Au2…Au1…Au2 angle being 82.15° (Figure 5). Adjacent tetramers in such chains also interact by N2-H2…N1[−1/2 + *x*, 1 − *y*, *z*] hydrogen bonds. The chain topology was determined in TOPOS, and the environment around the gold atoms was established, assuming that the gold and nitrogen atoms were the closest neighbors. The coordination numbers determined in this way were 7, 5, and 5 for the Au1, Au2, and Au3 atoms, respectively. In the coordination sphere of every gold(I) cation, there are two nitrogen atoms bound at 2.012(3) and 2.012(3) Å for Au1, 2.002(3) and 2.012(3) Å for Au2, and 2.017(3) and 2.017(3) Å for Au3. The chains are arranged into an *ab* layer mainly via non-covalent weak F…F interactions created between N11 amidinate anions. Finally, the 3D network is formed by F…F contacts formed by N1 amidinate anions from adjacent layers. It should be noted that this structure is maintained either by aurophilic and hydrogen bonds (Figure S2) in the chain or by F…F interchain contacts. The F…F contacts are the most numerous intermolecular interactions in the crystal network, and the red spots on the Hirshfeld surface indicate that some of those contacts are short [36,37].

**Figure 6:**
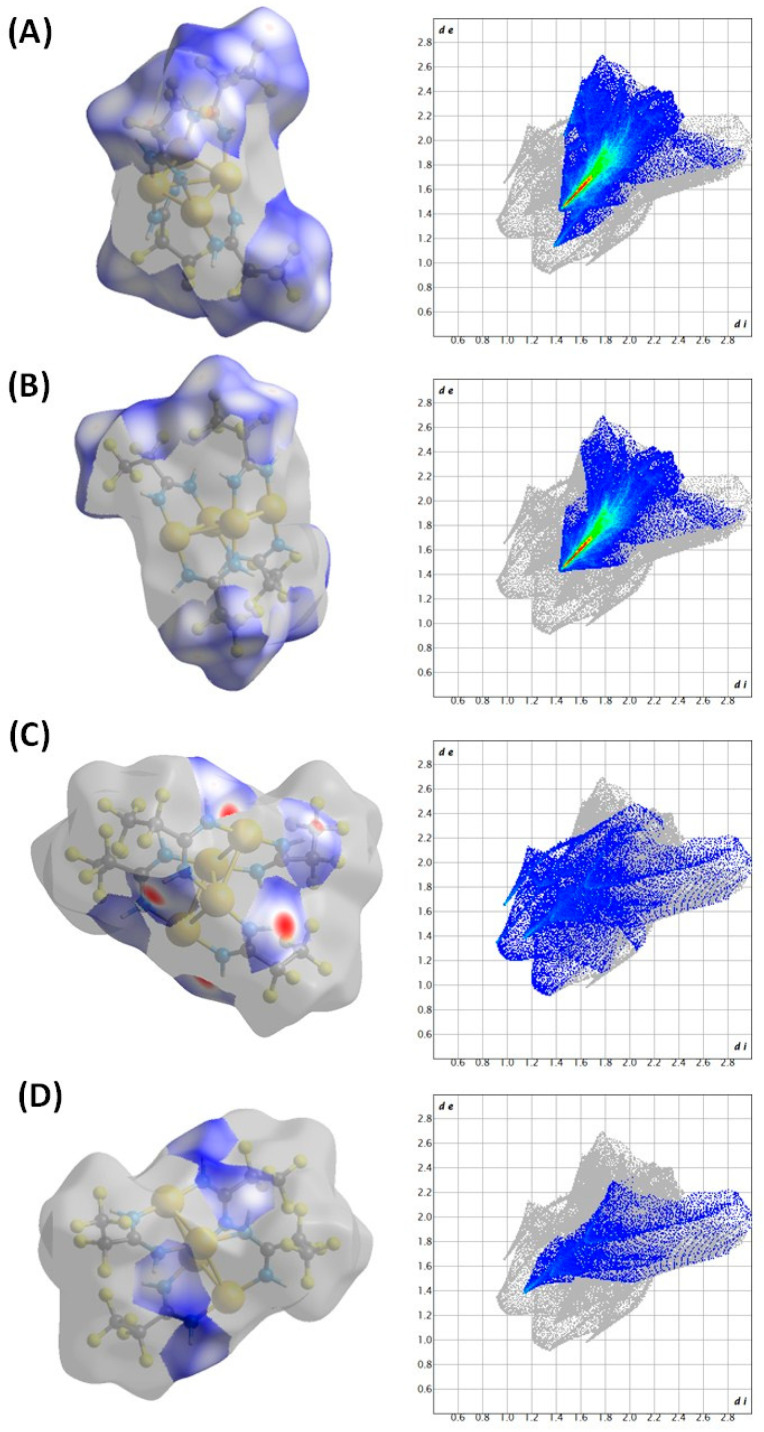
Hirshfeld surfaces (left) and fingerprints (right) of selected interactions created in the crystal network of [Au_4_(µ-AMDC_2_F_5_)_4_]_n_ (**1**): (**A**) for F…all (65.6%), (**B**) for F…F (53.1%), (**C**) for H…all (20.4%), (**D**) for H…F (9.5%). In brackets, a given surface area included as a percentage of the total surface area is shown.

Summarizing, the polymeric structure is maintained by aurophilic and hydrogen bonds (Figure 4 and Figure S2), whereas for interchain interactions, F…F contacts prevail, being the most numerous, with some short distances detected as it is evidenced by the red spots on the Hirshfeld surface (Figure 6, Figures S3 and S4). The presence of weak intermolecular interactions is advantageous in terms of application using the CVD method [Au_4_(µ-AMDC_2_F_5_)_4_]_n_ (**1**) and when introducing the compound via the GIS during the FEBID or FIBID process, which requires the use of volatile precursor molecules.

### 3.2. Infrared Spectra Analysis

**Figure 7:**
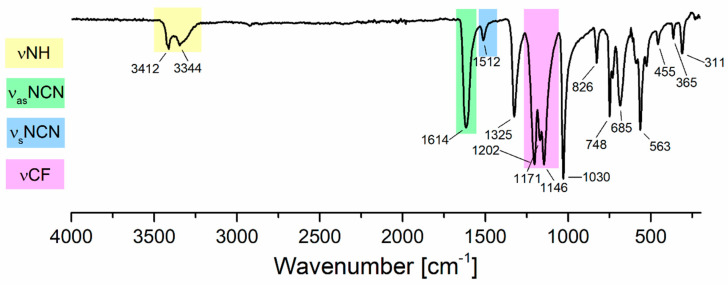
ATR-IR spectrum of the compound [Au_4_(µ-AMDC_2_F_5_)_4_]_n_ (**1**). The colored bands highlight the ranges of the particular vibrational modes: (νNH) (yellow), (ν_as_NCN) (green), (ν_s_NCN) (blue), (νCF) (pink).

In the infrared spectrum of the compound [Au_4_(µ-AMDC_2_F_5_)_4_]_n_ (**1**) (Figure 7), bands at 1614 cm^−1^ (**1**), characteristic of asymmetric stretching vibrations ν_as_NCN, as well as at 1512 cm^−1^, typical of symmetric ν_s_NCN stretching vibrations of the NCN group, were observed (Table 1), which confirmed the deprotonation of amidine and the formation of the amidinate complex. Moreover, the formation of the Au–N bond resulted in the appearance of a signal around 563 cm^−1^. The resolved crystal structure (Section 3.1) and EI MS (Section 3.4) results also indicated the formation of a gold(I) amidinate complex.

For the complex [Au_2_Cl_2_(HAMDC_2_F_5_)_2_]_n_ (**2**) (Figure S5), bands characteristic of the amidine molecule were observed in the spectrum, i.e., ν_as_NH_2_ at 3360; ν_s_NH_2_ 3233 cm^−1^; ν=NH at 3163 cm^−1^; νCN at 1678 cm^−1^; δNH_2_ 1611 cm^−1^. The coordination shifts of these signals relative to the free amidine were νNH_2_ → 129 cm^−1^; ν=NH → 33 cm^−1^; νCN → 14 cm^−1^; and δNH_2_ → 18 cm^−1^, confirming the coordination of this ligand. Moreover, the existence of signals for stretching vibrations, i.e., νAu–N at 565 cm^−1^ and νAu–Cl at 338 cm^−1^ [39], confirmed the formation of a bond between gold and the N-donor amidine as well as a of bond between chloride and the gold atoms. The TEM-EDX analysis of the complex (**2**) also confirmed the presence of chlorine atoms (Figure S6). Based on these data, we propose the formation of the chloro(amidine)gold(I) complex [Au_2_(HAMDC_2_F_5_)_2_Cl_2_]_n_ (**2**).

This data interpretation is crucial for the evaluation of the infrared result from the layers of compound (**1**) deposited on the silicon substrate (Section 3.5).

### 3.3. Thermal Analysis

**Figure 8:**
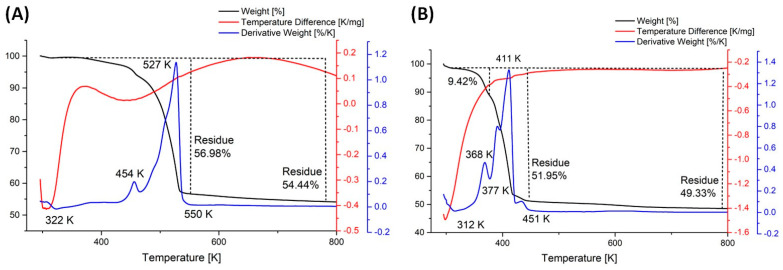
Thermogravimetric analysis (TGA) (black line), derivative thermogravimetry (DTG) (blue line), and differential thermal analysis (DTA) (red line) of the complex [Au_4_(µ-AMDC_2_F_5_)_4_]_n_ (**1**) (**A**) and [Au_2_Cl_2_(HAMDC_2_F_5_)_2_]_n_ (**2**) (**B**).

**Figure 9:**
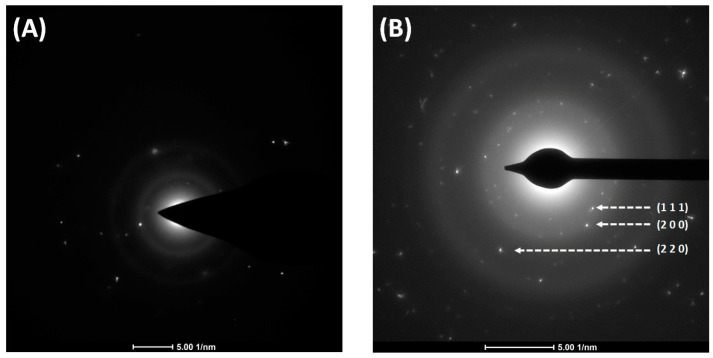
Transmission electron microscope (TEM) diffraction pattern of the TGA final residues of [Au_4_(µ-AMDC_2_F_5_)_4_]_n_ (**1**) (**A**) and [Au_2_Cl_2_(HAMDC_2_F_5_)_2_]_n_ (**2**) (**B**), with the diffraction pattern of gold marked in white.

Thermal analysis was performed to study the behavior of the complexes during heating under a nitrogen atmosphere and to determine the temperature of the final decomposition. These data revealed that the decomposition of complex (**1**) proceeded in a single main endothermic step. In the case of compound (**2**), two consecutive endothermic steps were observed (Figure 8, Table 2). The thermal process began at a temperature (T_i_) of ca. 317 K, with the maximum rate occurring at 527 K for (**1**) and 411 K for (**2**) (T_m_). The final decomposition temperature (T_f_) was 550 K (**1**) and 451 K (**2**), respectively (Table 2). The compounds (**1**) and (**2**) final residues are close to the theoretical content of gold in the respective molecules(Table 2). This indicated that for (**1**), the amidinates were lost. For complex (**2**), hydrogen chloride (377 K) was released in the first stage, amidinates detached in the second, and metallic gold formed as the final decomposition product. These results were confirmed by the TEM diffraction patterns of the formed residues, which revealed characteristic signals of metallic gold for both complexes (**1**) and (**2**) (Figure 9). Contrary to our previous results for the copper(I) and silver(I) amidinates [Cu_2_(µ-AMDC_2_F_5_)_2_] and [Ag_2_(µ-AMDC_2_F_5_)_2_], indicating that they are sources of metal carriers in the gas phase under atmospheric pressure in nitrogen [25], the gold(I) complexes (**1**) and (**2**) decomposed to the theoretically predicted amounts of metallic gold. That behavior can be justified by the different structures of the molecules.

### 3.4. Mass Spectra Analysis

The electron impact mass spectra (EI MS) of the complexes (**1**) and (**2**) were registered between 313 and 573 K. Based on these results, we could verify whether these compounds are capable of generating volatile metal carriers, check their sensitivity to low-energy electrons (70 eV), and study how the composition of the gas phase changed during heating under high vacuum (~10^−6^ mbar). It is important to note that during the FEBID or FIBID process in the SEM chamber, the conditions were quite similar to those of the EI MS experiments.

In the EI MS spectra, the metallated fragments were detected in relatively low temperatures, i.e., from 319 to 416 K for (**1**) and from 336 to 456 K for (**2**), which is promising for application in vapor deposition methods. These fragments achieved the following highest relative intensities: 100% RI from 326 K to 341 K and from 352 K to 403 K (**1**) and 63% RI at 343 K (**2**).

**Figure 10:**
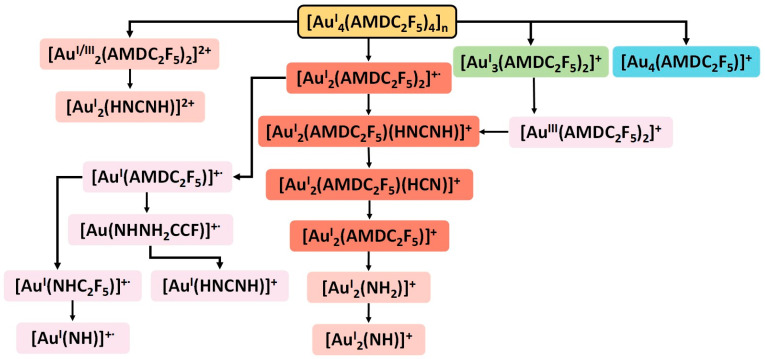
Fragmentation scheme for the metallated fragments of [Au_4_(µ-AMDC_2_F_5_)_4_]_n_ (**1**); assignment: blue—tetranuclear ions, green—trinuclear ions, red—dinuclear ions, pink—mononuclear ions (brighter fields—ions occurring in small quantities).

In the case of the compound [Au_4_(µ-AMDC_2_F_5_)_4_]_n_ (**1**), the high %RI of the ion [Au_2_(NHNHCC_2_F_5_)_2_]^+•^ (RI = 100%) (Figure S8) indicates its stability and not highly efficient low-energy electron-induced fragmentation. Moreover, row of dinuclear fragments were present as dominant under the process conditions (Figure 10). This suggests a thermal and electron-induced reaction resulting in the breaking of the polymer chains. However, based on the [Au_3_(NHNHCC_2_F_5_)_2_]^+^ and [Au_4_(NHNHCC_2_F_5_)]^+^ ions, the formation of higher mass fragments cannot be excluded. Due to the limited measuring range up to m/z 1100, it was not possible to record them.

The EI MS results for [Au_2_Cl_2_(HAMDC_2_F_5_)_2_]_n_ (**2**) showed that the compound released HCl upon heating (electron-induced ions [HCl]^+•^ and [H_2_Cl]^+^ were detected) and converted to gold(I) amidinate, and fragments were obtained, as observed in the fragmentation of compound (**1**) (Figure S7, Table S4). Moreover, the ion [Au_2_(NHNHCC_2_F_5_)_2_]^+•^ confirmed amidine deprotonation and amidinate formation. The low %RI for the Au-AMDC_2_F_5_ fragment and high %RI for the [Au(NH)]^+•^ (RI_max_ = 63%) indicated an efficient electron-induced fragmentation. The signals m/z 87 and 88 (Table S4) are indicative of an impurity in the form of [Au(tht)Cl], resulting from the volatile tetrahydrotiophene used during synthesis.

Moreover, the signals characteristic for the ions, e.g., [CF_2_]^+^, [CF_3_]^+^, [CF_2_CN]^+^, [C_2_F_4_CN]^+^, [NHNH_2_CC_2_F_5_]^+•^, indicated the fragmentation of compounds (**1**), (**2**) and are related to a reaction pathway that can transform the amidinate unit to volatile fluorinated molecules.

Summarizing, the [Au_4_(µ-AMDC_2_F_5_)_4_]_n_ (**1**) and [Au_2_Cl_2_(HAMDC_2_F_5_)_2_]_n_ (**2**) complexes can be promising for use in the CVD, FEBID, and FIBID processes because the effective generation of volatile metal carriers, e.g., gold amidinates in EI MS was observed at relatively low temperatures (from 319 K (**1**) and 339 K (**2**)). Therefore, it can be assumed that the investigated compounds can be introduced into the microscope chamber via the GIS system at a temperature similar to that used for the commercial platinum precursor [Pt(µ^5^-CpMe)Me_3_], which is about 333 K [6,18]. Moreover, [Au_2_Cl_2_(HAMDC_2_F_5_)_2_]_n_ (**2**) decomposed to gold(I) amidinate and fragmented in the carbon-free fragment [Au(NH)]^+•^ with high efficiency. This is important because one of the problems associated with FEBID is the carbon contamination of structures [6,18]. Moreover, the complexes (**1**) and (**2**) are sensitive to low-energy electron interactions. Therefore, they seem to be promising candidates for CVD applications and FEBID or FIBID tests.

### 3.5. Sublimation Experiments

Sublimation is a desired property for complexes that are used in vapor deposition methods such as CVD and FEBID or FIBID. Sublimation experiments were carried out to verify the volatility of the new gold(I) complexes (**1**) and (**2**) and determine the sublimation temperature of entire compound molecules. These experiments were carried out in a classical glass sublimator under a pressure of 10^−2^ mbar with a special holder for a silicon substrate. Moreover, they allowed for obtaining layers of compound (**1**), which was then studied in terms of interaction with a high-energy electron beam (Section 3.7).

**Figure 11:**
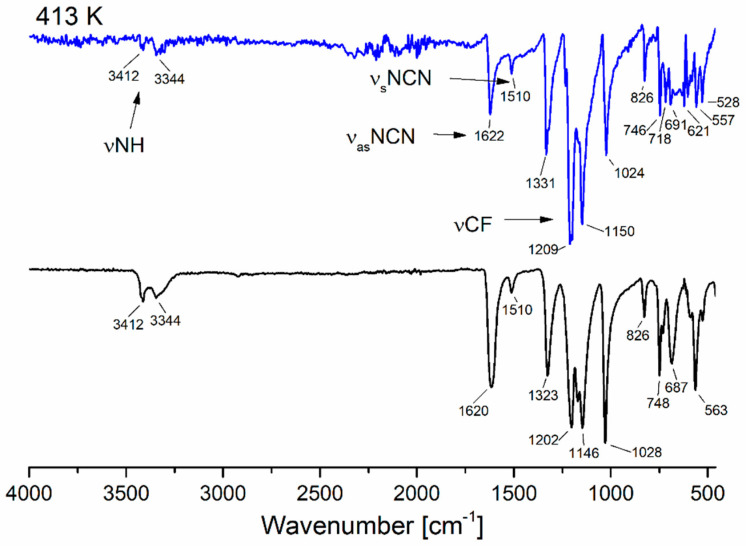
Infrared spectra of the compound [Au_4_(µ-AMDC_2_F_5_)_4_]_n_ (**1**) before (black) and after sublimation (blue) at 413 K (p = 10^−2^ mbar).

The infrared spectrum of a [Au_4_(µ-AMDC_2_F_5_)_4_]_n_ (**1**) thin layer deposited on a silicon substrate was very similar to that obtained in the solid phase and exhibited the bands νNH, ν_as_NCN, ν_s_NCN, and νCF, which are characteristic of coordinated amidinate (Figure 11). There were some additional bands around 800–500 cm^−1^, probably resulting from some primary polymeric structure rearrangement. This fact does not affect the conclusion that the complex sublimes at a temperature of 413 K. In the case of the compound [Au_2_Cl_2_(HAMDC_2_F_5_)_2_]_n_ (**2**), the sublimation experiment was conducted up to 353 K. The differences in the shape and position of the bands in the spectra of the original complex and the formed layer during the process (Figure S9) indicate that decomposition products dominated in the layer. However, signals characteristic of gold(I) amidinate were also observed in the spectrum due to decomposition of the complex (**2**), but their intensity was small. Furthermore, the SEM-EDX spectra analysis of the deposited layer showed Cl, F, N, and C, which confirmed that compound (**2**) decomposed into organic species (Figure S10), while the negligible intensity of the Au signal indicated gold(I) amidinate formation. On the other hand, the recorded TEM diffraction pattern of non-volatile residue revealed that the final product of the decomposition was gold nanoparticles (Figure S11).

The compounds’ volatility is usually discussed in relation to the molecular weight and the interactions occurring in the solid state. The good volatility of complex (**1**) can be justified by the polymeric structure maintained by the aurophilic and hydrogen bonds in the chain and the F…F contacts dominating in the crystal network. The aurophilic interactions (2.5–3.5 Å) are a significant stabilization factor responsible for an evaporation temperature increase [18]. However, in compound (**1**), those bonds are short inside the tetramers, whereas they are much longer (3.361 Å) and weaker between adjacent tetrameric motifs. Moreover, the huge number of intermolecular F…F contacts providing significant electrostatic repulsion also affects volatility. Hence, both factors—weak aurophilic bonds between tetramers and interactions between partially negatively polarized fluorine atoms—are responsible for the lowering of the evaporation temperature [23].

The advantage of [Au_4_(µ-AMDC_2_F_5_)_4_]_n_ (**1**) with N,N-donor ligands is its slightly lower evaporation temperature compared to that of the O,O-donor carboxylate (423 K, 10^−1^ mbar), which is a well-known FEBID precursor [Ag_2_(µ-O_2_CC_2_F_5_)_2_] (76 at.% Ag) [6,40].

### 3.6. Variable-Temperature Infrared Spectroscopy (VT IR)

The compound [Au_2_Cl_2_(HAMDC_2_F_5_)_2_]_n_ (**2**) was studied using the VT IR method to determine the composition of the gas phase formed during the heating of the complex. Moreover, we could verify that the studied complex was capable of generating volatile metal carriers under higher pressure (10^−1^ mbar) than during the sublimation attempt and under which it decomposed (Section 3.3 and Section 3.5).

**Figure 12:**
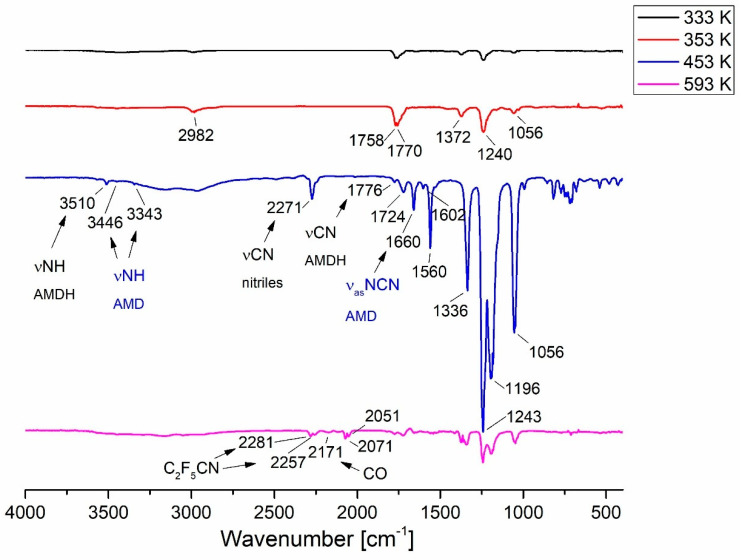
VT IR spectra of [Au_2_Cl_2_(HAMDC_2_F_5_)_2_]_n_ (**2**) in the temperature range of 333–593 K (p = 10^−1^ mbar).

In the spectra registered for the compound [Au_2_Cl_2_(HAMDC_2_F_5_)_2_]_n_ (**2**) (Figure 12), the first volatile products formed upon heating were recorded at 333 K. At this temperature, a band characteristic for HCl molecules was detected at 2982 cm^−1^. This indicated that for the complex [Au_2_Cl_2_(HAMDC_2_F_5_)_2_]_n_ (**2**), the generation of volatile gold carriers is driven by a thermal process resulting in the release of hydrogen chloride, amidine deprotonation, and the formation of gold(I) amidinate [Au_4_(µ-AMDC_2_F_5_)_4_]_n_ (**1**), which was also deduced from the EI MS studies and the sublimation experiments (Section 3.4 and Section 3.5). The VT IR spectra at the temperature of 433 K and 453 K showed the bands νNH (3446, 3343 cm^−1^), and ν_as_NCN (1660 cm^−1^) (Figure 12), characteristic for the gold(I) amidinate. However, the occurrence of the bands characteristic for free amidine, i.e., νNH (3510 cm^−1^), νCN (1776 cm^−1^), and νC≡N (2271 cm^−1^), in the temperature range of 373–453 K, suggest that a decomposition products’ mixture was formed simultaneously (Figure 12).

In summary, it was confirmed that the amidine complex [Au_2_Cl_2_(HAMDC_2_F_5_)_2_]_n_ (**2**) generates metal carriers in the gas phase in the form of gold(I) amidinate and can be considered for use in vapor deposition processes.

### 3.7. SEM/EDX Observation and TEM/EDX Studies of [Au_4_(µ-AMDC_2_F_5_)_4_]_n_ (**1**) Adsorbed Thin Layers

**Figure 13:**
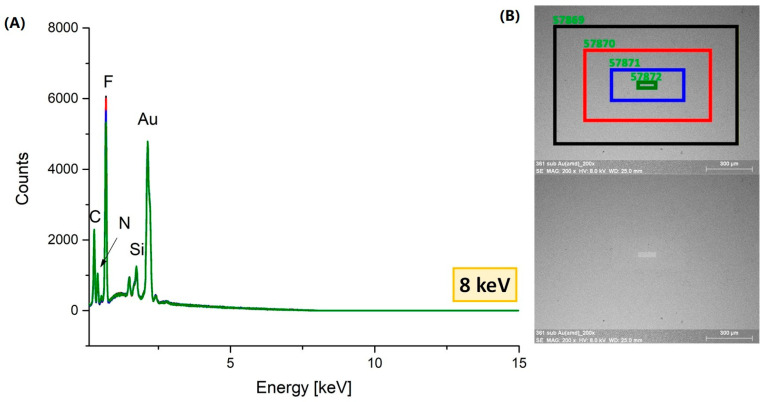
EDX spectra (8 keV) of sublimed [Au_4_(µ-AMDC_2_F_5_)_4_]_n_ (**1**) on a Si(111) substrate (Mag = 200×) (**A**). Top view of SEM images of irradiated areas of the sublimed film (**B**); top image: marked areas corresponding to the EDX plots; bottom image: morphological changes after irradiation; black, red, blue, and green rectangles—orderly with largest to smallest area of scanning, the colors correspond to changes in signals intensity in the EDX spectrum.

**Figure 14:**
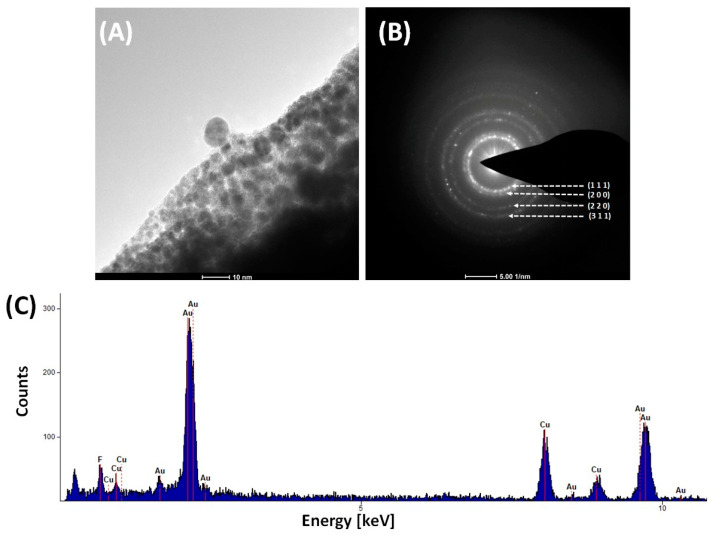
TEM imaging for [Au_4_(µ-AMDC_2_F_5_)_4_]_n_ (**1**): image of the sample after several seconds of interaction with high-energy (200 keV) electrons (**A**), TEM diffraction pattern (**B**), TEM-EDX spectrum (**C**).

To assess the sensitivity of the compounds to high-energy electrons and their usefulness in the FEBID and FIBID methods, a scanning electron microscope (SEM) and a transmission electron microscope (TEM) were used. These experiments are important to evaluate the precursor’s initial effectiveness in the FEBID and FIBID process and provide insights for the design of new precursors. The sensitivity of thin layers of complex (**1**) resublimated on a silicon substrate (Section 3.5) was studied using SEM/EDX at 8 and 20 keV energy. EDX spectra were recorded during the SEM analysis. The scanning area was reduced, which caused an increase in the dose of electrons. Moreover, TEM (200 keV) measurements were performed for compound (**1**). The EDX spectra recorded for each area showed that changes in the intensity of the signals characteristic of individual elements (C, N, and Au) were observed when irradiation at 8 keV was performed (Figure 13 and Figure S12). Only a decrease in the fluorine signal intensity was observed. Based on these results and the morphological changes observed in SEM, we propose that complex (**1**) is sensitive to a high-energy electron beam (Figure 14). With the use of 20 keV, the changes in signal intensity for individual elements (C, N, F, and Au) remained at the same level, and no morphological changes in the SEM image were observed (Figure S12).

Moreover, the mentioned vaporized layer of compound (**1**) was heated at 613 K, following the thermal analysis, under conditions close to those of the CVD process. Notably, characteristic signals for Au and C were observed, while no signals for F and N were detected in the EDX spectrum (Figure S13), which is an encouraging result for the potential application of compound (**1**) in the CVD process. These results suggest a more efficient thermal removal of the organic ligand from the surface relative to the electron irradiation. However, this hypothesis requires confirmation through further experiments.

In the TEM imaging (200 keV) that was performed for [Au_4_(µ-AMDC_2_F_5_)_4_]_n_ (**1**), changes in the structure of the sample were observed. After a few seconds of irradiation, the TEM images revealed grains surrounded by amorphous carbon (Figure 14A). The diffraction patterns confirmed the formed material’s crystallinity and gold nanoparticle formation (Figure 14). The TEM EDX spectra from TEM showed signals characteristic of gold, fluorine, and carbon, but no nitrogen signal was detected. It should be noted that the TEM mesh was covered with a carbon membrane (Table 3). As a result of the interaction of the complex (**1**) with the electron beam (200 keV), a decomposition product formed, which was identified as crystalline gold. The absence of a signal from nitrogen indicates that the Au-N bond was broken under the conditions of the TEM observations. Moreover, the fluorine signal suggests that a carbon fluoride matrix was formed.

In summary, the microscopic observations confirmed that compound (**1**) demonstrates sensitivity to high-energy electrons and can potentially be tested under experimental FEBID or FIBID conditions. It should be noted that the decomposition of complex (**1**) strongly depends on the beam power and electron dose.

## 4. Conclusions

Synthesis methods of new gold(I) complexes based on the fluorinated amidine with the formulas [Au_4_(µ-AMDC_2_F_5_)_4_]_n_ (**1**) and [Au_2_Cl_2_(HAMDC_2_F_5_)_2_]_n_ (**2**) were developed. For [Au_4_(µ-AMDC_2_F_5_)_4_]_n_ (**1**), single-crystal X-ray diffraction studies revealed the formation of a stable coordination polymer composed of [Au_4_(µ-AMDC_2_F_5_)_4_] (**1**) tetramers and demonstrated the presence of bridging amidinates as well as aurophilic bonds in the chain. Additionally, the IR and EI MS spectra analysis confirmed the amidinate formation and the coordination of gold by nitrogen atoms. The relatively low evaporation temperature, 413 K (p = 10^−2^ mbar), can be related to the structural features—aurophilic interactions, which are short in the tetramer and much longer and weaker between adjacent repeating motifs—and the dominant role of weak repulsive F...F intermolecular interactions between adjacent chains, confirmed by Hirshfeld surface analysis. In the case of the complex [Au_2_Cl_2_(HAMDC_2_F_5_)_2_]_n_ (**2**), the EI MS results indicated the formation of dinuclear gold amidinate fragments released upon the thermal loss of HCl. The thermal analysis data indicated that the compounds decomposed at the atmospheric pressure, with loss of amidinate for (**1**) and (**2**) and hydrogen chloride in the first step for (**2**). On the other hand, the EI MS spectra analysis showed that both gold(I) complexes were the source of metal in the gas phase, even at the temperature of 319 K (p = 10^−6^ mbar). This fact suggests that the studied complexes could be introduced into the SEM microscope chamber via the GIS system. The VT IR spectra confirmed the release of HCl from [Au_2_Cl_2_(HAMDC_2_F_5_)_2_]_n_ (**2**) and the formation of gold(I) amidinate over the temperature range of 433–453 K. [Au_4_(µ-AMDC_2_F_5_)_4_]_n_ (**1**) sublimation occurs at 413 K (p = 10^−2^ mbar), which is similar to what observed for the sublimation of the FEBID precursor [Ag_2_(µ-O_2_CC_2_F_5_)_2_]. These results indicate that the new gold(I) amidinate is a metal source in the gas phase and can be tested under typical CVD conditions. Based on the study of the complex’ interactions with high-energy electrons, it can be concluded that the compound [Au_4_(µ-AMDC_2_F_5_)_4_]_n_ (**1**) is sensitive to high-energy electron exposure (8 and 200 keV). Moreover, [Au_4_(µ-AMDC_2_F_5_)_4_]_n_, unlike previously tested gold(I) and gold(III) complexes, is air-stable and user-friendly, shows satisfactory volatility, is susceptible to an electron beam, and can be considered for practical use in the FEBID and FIBID processes.

## Figures and Tables

**Table 1:** Selected IR absorption bands (cm^−1^) of the studied compounds (**1**) and (**2**).

Compound	νNH	νNH_2_	ν=NH	νC=N	δNH_2_	ν_as_NCN	v_s_NCN	vAu-N
[Au_4_(µ-AMDC_2_F_5_)_4_]_n_ (**1**)	3412 3344	─	─	─	─	1614	1512	563
[Au_2_Cl_2_(HAMDC_2_F_5_)_2_]_n_ (**2**)	─	3233	3163	1678	1611	─	─	565
HAMDC_2_F_5_	─	3362	3130	1664	1593	─	─	─

The band of vAu-Cl = 338 cm^−1^ in the [Au_2_Cl_2_(HAMDC_2_F_5_)_2_]_n_ (**2**) spectrum.

**Table 2:** Thermal analysis results.

Complex	Heat Effect	Temperature [K]			Residue [%]	
		$T_{i}$	$T_{m}$	$T_{f}$	Found [%]	Calc. [%]
[Au_4_(µ-AMDC_2_F_5_)_4_]_n_ (**1**)	Endo	322	527	550	56.98	55.01 (Au)
[Au_2_Cl_2_(HAMDC_2_F_5_)_2_]_n_ (**2**)	Endo Endo	312 377	368 411	377 451	51.95	49.99 (Au)

*T_i_*, initial temperature; *T_m_*, maximum temperature; *T_f_*, final temperature.

**Table 3:** TEM-EDX data for the complex [Au_4_(µ-AMDC_2_F_5_)_4_]_n_ (**1**).

Element	Atomic Content of the Element [%at.] after Irradiation	Atomic Content of the Element [%at.] of the Complex
	[Au_4_(µ-AMDC_2_F_5_)_4_]_n_ (1)	
Au	83.02	1
F	13.13	5
C	3.84 *	3
N	─	2

* TEM grid covered with carbon.

## Data Availability

The data presented in this study are available on request from the corresponding authors.

## Supplementary Materials

The following supporting information can be downloaded at: https://www.mdpi.com/article/10.3390/molecules30010146/s1, Table S1 Crystal data and structure refinement for the compound [Au_4_(µ-AMDC_2_F_5_)_4_]_n_ (**1**); Table S2 Bond lengths [Å] and angles [°] for the compound [Au_4_(µ-AMDCC_2_F_5_)_4_]_n_ (**1**); Figure S1 The asymmetric part of structure of [Au_4_(µ-AMDC_2_F_5_)_4_]_n_ (**1**) with ellipsoids at the level of 30% probability (yellow—gold atoms; blue—nitrogen atoms; gray—carbon atoms; green—fluorine atoms); Figure S2 Crystal structure projected in the ab plane of [Au_4_(µ-AMDC_2_F_5_)_4_]_n_ (**1**) (hydrogen bonds H...N are marked with a red dashed line); Figure S3 Hirshfeld surfaces (left) and fingerprints (right) of selected interactions created in the crystal network of [Au_4_(µ-AMDC_2_F_5_)_4_]_n_ (**1**): (A) for F…H (9.2%), (B) for H…H (5.3%), (C) for Au…F (3.4%), (D) for Au…H (3.4%). In brackets, a given surface area included as a percentage of the total surface area is shown; Figure S4 Hirshfeld surfaces (left) and fingerprints (right) of selected interactions created in the crystal network of [Au_4_(µ-AMDC_2_F_5_)_4_]_n_ (**1**): (A) for H…Au (2.7%), (B) for N…H (2.6%), (C) for H…N (2.6%), (D) for Au…Au (2.3%). In brackets, a given surface area included as a percentage of the total surface area is shown; Figure S5 ATR-IR spectrum for the compound [Au_2_Cl_2_(HAMDC_2_F_5_)_2_]_n_ (**2**) (blue), HAMDC_2_F_5_ (red), and [Au(tht)Cl] (black); Figure S6 Results of TEM-EDX analysis obtained for the complex [Au_2_Cl_2_(HAMDC_2_F_5_)_2_]_n_ (**2**) after a few seconds of interaction with a focus high-energy electron beam (200 keV), (A)—TEM image, (B) TEM-EDX spectrum, and (C) atomic content of the element; Table S3 Electron impact mass spectrometry (EI MS) results for the compound [Au_4_(µ-AMDC_2_F_5_)_4_]_n_ (**1**); Table S4 Electron impact mass spectrometry (EI MS) results for the compound [Au_2_Cl_2_(HAMDC_2_F_5_)_2_]_n_ (**2**); Figure S7 Fragmentation scheme for the most important ions of [Au_2_Cl_2_(HAMDC_2_F_5_)_2_]_n_ (**2**) (HAMD–NHNH_2_CC_2_F_5_; AMD–(NH)_2_CC_2_F_5_)), assignment: grey—non-metalled ions, red—dinuclear ions, pink—mononuclear ions; Figure S8 EI MS spectra, where the [Au_2_(AMD)_2_]^+·^ ion achieved relative intensity at the level of 100%; Figure S9 Infrared spectra for the compound [Au_2_Cl_2_(HAMDC_2_F_5_)_2_]_n_ (**2**) before (black) and after sublimation (blue) at 353 K (p = 10^−2^ mbar); Figure S10 EDX spectra (20 keV) of product adsorbed on a Si(111) substrate during the sublimation test of [Au_2_Cl_2_(HAMDC_2_F_5_)_2_]_n_ (**2**); Figure S11 Transmission electron microscope image for gold nanoparticles formed after sublimation process for the complex [Au_2_Cl_2_(HAMDC_2_F_5_)_2_]_n_ (**2**) (A), TEM diffraction pattern (B), and energy-dispersive X-ray spectroscopy (EDX) spectrum (TEM grid covered with carbon) (C); Figure S12 EDX spectra (20 keV) of sublimed [Au_4_(µ-AMDC_2_F_5_)_4_]_n_ (**1**) on a Si(111) substrate (Mag = 200×) (A). Top view SEM images of irradiated areas of the sublimed film (B): top: with marked areas corresponding to the EDX plots; bottom: morphological changes after irradiation; black, red, blue, and green rectangles – orderly with largest to smallest area of scanning, the colors correspond to changes in signals intensity in the EDX spectrum; Figure S13 SEM images of vaporized layer of the compound [Au_4_(µ-AMDC_2_F_5_)_4_]_n_ (**1**) after heat treatment at 613 K: Mag = 10,000 x—(A); Mag = 50,000 x—(B); Mag = 100,000 x—(C). SEM image—(D) and EDX spectrum—(E) of studied area.
