# Supplementary material for: New Gold(I) Complexes as Potential Precursors for Gas-Assisted Methods: Structure, Volatility, Thermal Stability, and Electron Sensitivity
Source: Molecules. 2025 Jan 2;30(1):146. doi: 10.3390/molecules30010146 (PMC11721683; doi:10.3390/molecules30010146)
Supplement: Supplementary file 1 [file molecules-30-00146-s001.zip › final_SI_Au(AMD)_31.12.2024_ISz_ABK_TM.pdf]

New Gold(I) Complexes as Potential Precursors for Gas-Assisted Methods: Structure, Volatility, Thermal Stability, and Electron Sensitivity

Aleksandra Butrymowicz-Kubiak, Tadeusz M. Muzioł, Piotr Madajski, Iwona B. Szymańska**

Faculty of Chemistry, Nicolaus Copernicus University in Toruń, Gagarina 7, 87-100 Toruń, Poland

*Corresponding authors: Iwona B. Szymańska: Tel.: +48 566114317; e-mail address: pola@umk.pl Tadeusz M. Muzioł, Tel.: +48-566114976; e-mail address: tmuziol@chem.umk.pl

Table S1. Crystal data and structure refinement for the compound $[Au_4(\mu\text{-}AMDC_2F_5)_4]_n$ (**1**).

**Identification code**	**(1)**
**Empirical formula**	C12 H8 Au4 F20 N8
**Formula weight**	1432.13
**Temperature [K]**	100(2)
**Wavelength [Å]**	0.71073
**Crystal system, space group**	Monoclinic, I2/a
**Unit cell dimensions [Å] and [°]**	a = 8.99189(19) α = 90<br>b = 12.6776(3) β = 98.034(2)<br>c = 22.7927(5) γ = 90
**Volume [Å^3]**	2572.77(10)
**Z, Calculated density [Mg×m^{-3}]**	4, 3.697
**Absorption coefficient [mm^{-1}]**	22.906
**F(000)**	2528
**Crystal size [mm^3]**	0.150 x 0.040 x 0.020
**Theta range for data collection [°]**	2.796 to 26.370
**Limiting indices**	-11<=h<=10<br>-15<=k<=15<br>-28<=l<=28
**Reflections collected/unique**	15417
**Completeness [%] to theta [°]**	99.6 %
**Absorption correction**	Gaussian
**Max. and min. transmission**	0.854 and 0.277
**Refinement method**	Full-matrix least-squares on F^2
**Data/restraints/parameters**	2625 / 0 / 200
**Goodness-of-fit on F^2**	1.035
**Final R Indices [I>2sigma(I)]**	R1 = 0.0145, wR2 = 0.0355
**R indices (all data)**	R1 = 0.0171, wR2 = 0.0364
**Largest diff. peak and hole [eÅ^{-3}]**	1.184 and -0.916

Table S2. Bond lengths [Å] and angles [°] for the compound $[Au_4(\mu\text{-}AMDCC_2F_5)_4]_n$ (**1**).

Au(1)-N(2)#1	2.012(3)
Au(1)-N(2)	2.012(3)
Au(1)-Au(2)#1	3.03500(14)
Au(1)-Au(2)	3.03506(14)
Au(1)-Au(3)	3.3288(2)
Au(1)-Au(2)#2	3.36078(18)
Au(1)-Au(2)#3	3.36078(18)
Au(2)-N(12)	2.002(2)
Au(2)-N(1)	2.012(2)
Au(2)-Au(3)	3.04568(14)
Au(3)-N(11)#1	2.019(2)
Au(3)-N(11)	2.019(2)
N(2)#1-Au(1)-N(2)	177.79(14)
N(2)#1-Au(1)-Au(2)#1	78.40(7)
N(2)-Au(1)-Au(2)#1	102.84(7)
N(2)#1-Au(1)-Au(2)	102.84(7)
N(2)-Au(1)-Au(2)	78.40(7)
Au(2)#1-Au(1)-Au(2)	113.925(6)
N(2)#1-Au(1)-Au(3)	91.11(7)
N(2)-Au(1)-Au(3)	91.11(7)
Au(2)#1-Au(1)-Au(3)	56.963(3)
Au(2)-Au(1)-Au(3)	56.962(3)
N(2)#1-Au(1)-Au(2)#2	74.07(7)
N(2)-Au(1)-Au(2)#2	104.20(7)
Au(2)#1-Au(1)-Au(2)#2	147.724(4)
Au(2)-Au(1)-Au(2)#2	88.653(3)
Au(3)-Au(1)-Au(2)#2	138.924(3)
N(2)#1-Au(1)-Au(2)#3	104.20(7)
N(2)-Au(1)-Au(2)#3	74.07(7)
Au(2)#1-Au(1)-Au(2)#3	88.652(3)
Au(2)-Au(1)-Au(2)#3	147.724(4)
Au(3)-Au(1)-Au(2)#3	138.924(3)
Au(2)#2-Au(1)-Au(2)#3	82.152(6)
N(12)-Au(2)-N(1)	174.93(10)
N(12)-Au(2)-Au(1)	103.69(7)
N(1)-Au(2)-Au(1)	81.17(7)

N(12)-Au(2)-Au(3)	81.23(7)
N(1)-Au(2)-Au(3)	99.66(7)
Au(1)-Au(2)-Au(3)	66.381(5)
N(12)-Au(2)-Au(1)#2	82.20(7)
N(1)-Au(2)-Au(1)#2	99.25(7)
Au(1)-Au(2)-Au(1)#2	91.346(4)
Au(3)-Au(2)-Au(1)#2	147.922(4)
N(11)#1-Au(3)-N(11)	170.06(15)
N(11)#1-Au(3)-Au(2)#1	77.88(7)
N(11)-Au(3)-Au(2)#1	107.77(7)
N(11)#1-Au(3)-Au(2)	107.77(7)
N(11)-Au(3)-Au(2)	77.88(7)
Au(2)#1-Au(3)-Au(2)	113.312(7)
N(11)#1-Au(3)-Au(1)	94.97(7)
N(11)-Au(3)-Au(1)	94.97(7)
Au(2)#1-Au(3)-Au(1)	56.655(3)
Au(2)-Au(3)-Au(1)	56.656(3)
C(2)-N(1)-Au(2)	125.1(2)
Au(2)-N(1)-H(1)	117.5
C(2)-N(2)-Au(1)	129.3(2)
Au(1)-N(2)-H(2)	115.4
C(12)-N(11)-Au(3)	128.5(2)
Au(3)-N(11)-H(11)	115.8
C(12)-N(12)-Au(2)	125.8(2)
Au(2)-N(12)-H(12)	117.1

Symmetry transformations used to generate equivalent atoms:

#1 -x+1/2,y,-z+1   #2 -x+1,-y+1,-z+1   #3 x-1/2,-y+1,z

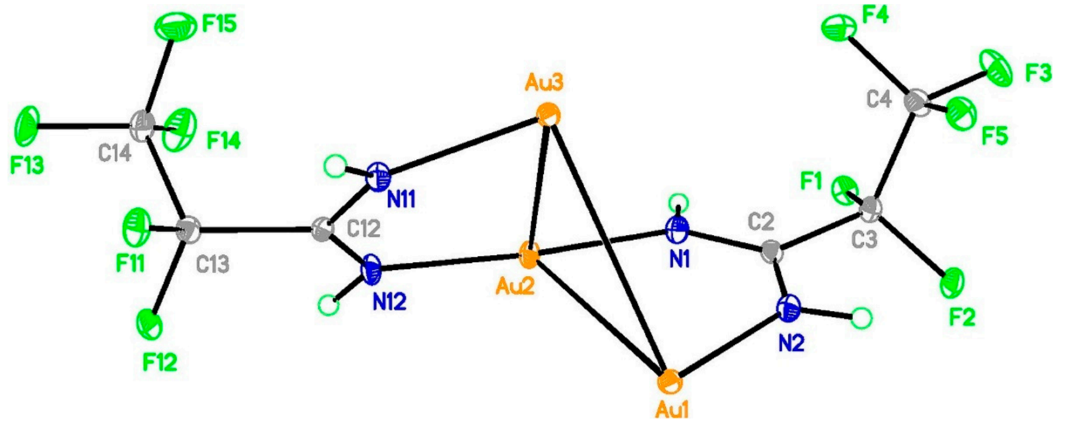

Figure S1. The asymmetric part of structure of [Au_4(μ-AMDC_2F_5)$_4$]$_n$ (**1**) with ellipsoids at the level of 30% probability (yellow – gold atoms; blue – nitrogen atoms; gray – carbon atoms; green – fluorine atoms).

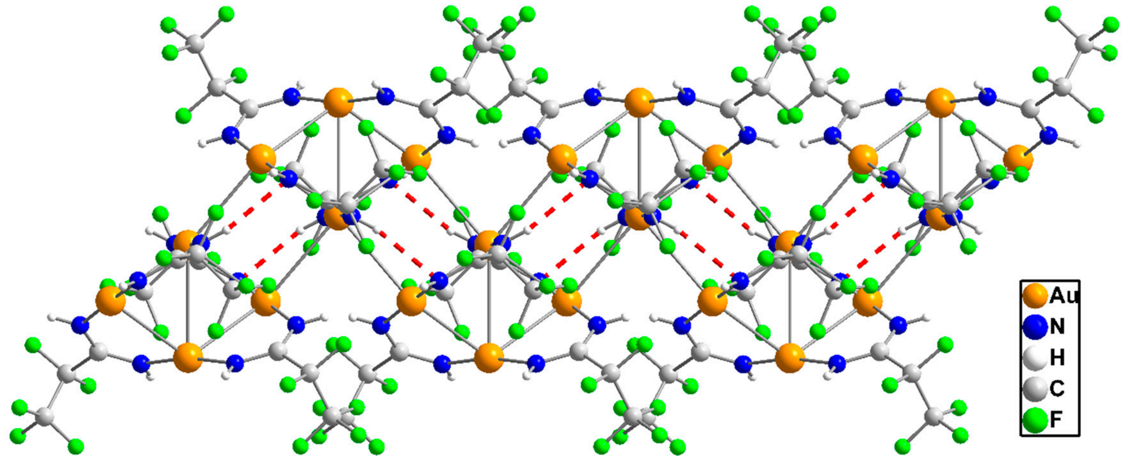

Figure S2. Crystal structure projected in the *ab* plane of [Au_4(μ-AMDC_2F_5)$_4$]$_n$ (**1**) (hydrogen bonds H...N are marked with a red dashed line).

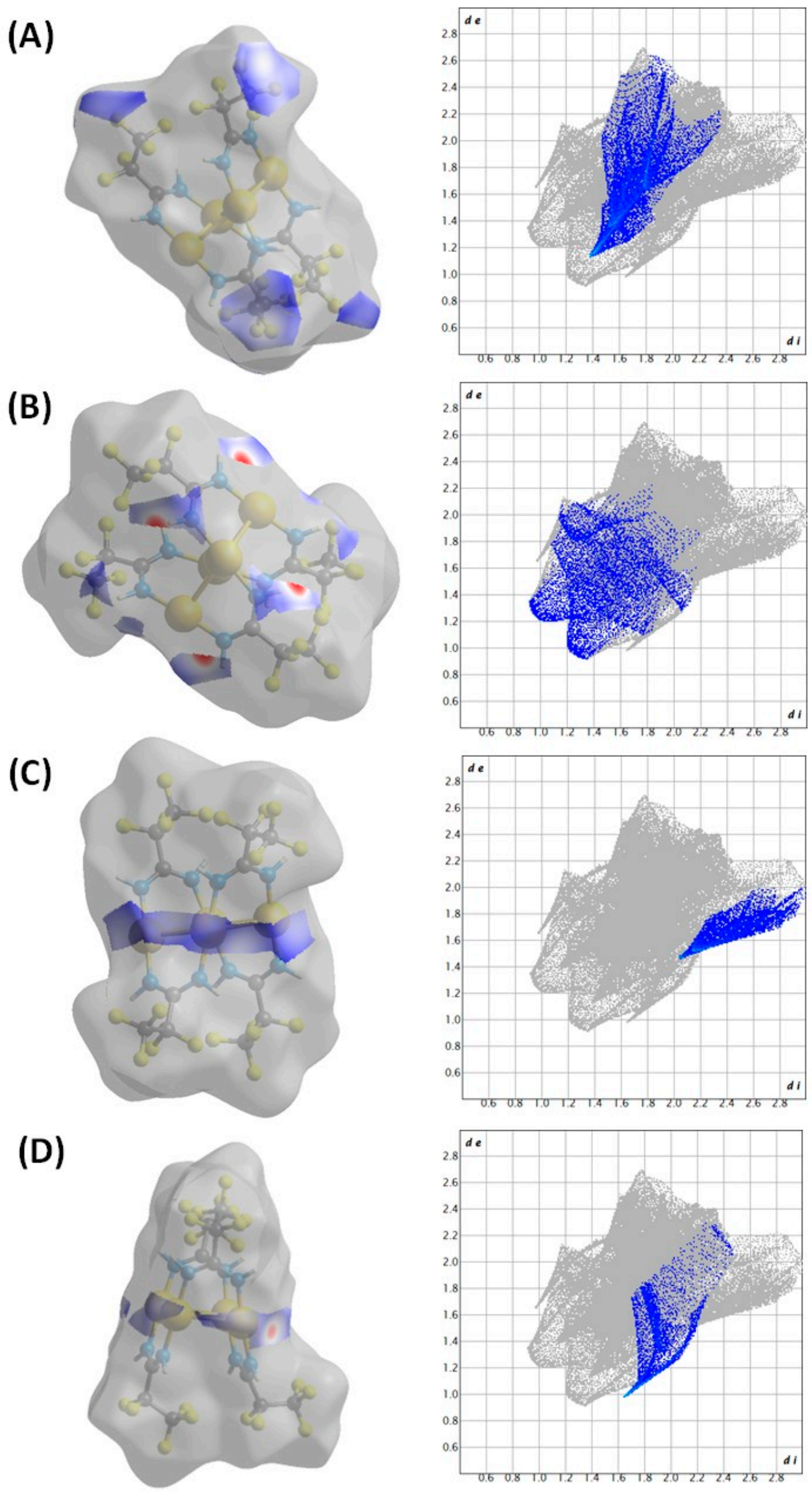

Figure S3. Hirshfeld surfaces (left) and fingerprints (right) of selected interactions created in the crystal network of $[Au_4(\mu\text{-}AMDC_2F_5)_4]_n$ (**1**): (A) for F…H (9.2%), (B) for H…H (5.3%), (C) for Au…F (3.4%), (D) for Au…H (3.4%). In brackets, a given surface area included as a percentage of the total surface area is shown.

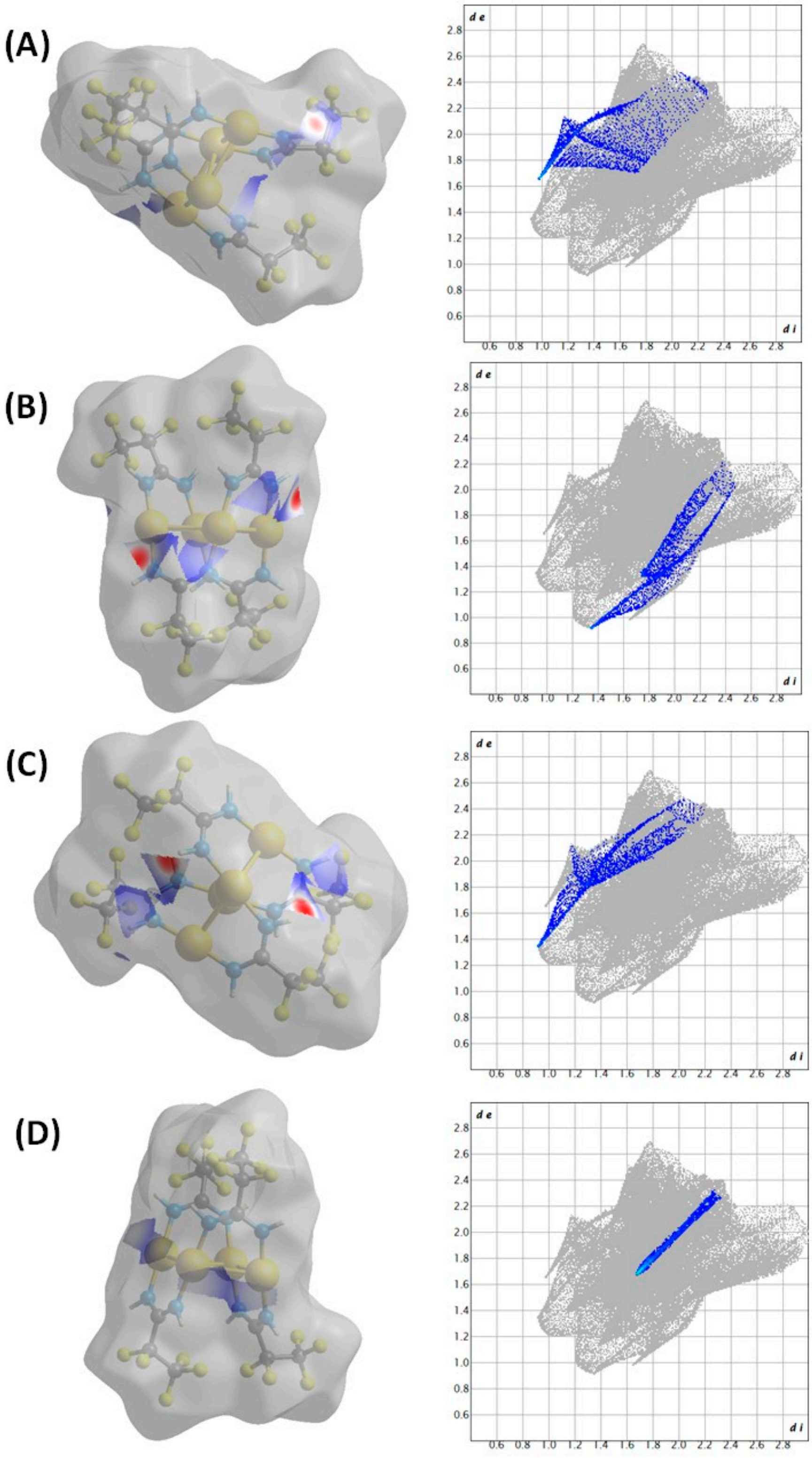

Figure S4. Hirshfeld surfaces (left) and fingerprints (right) of selected interactions created in the crystal network of $[Au_4(\mu\text{-}AMDC_2F_5)_4]_n$ (**1**): (A) for H…Au (2.7%), (B) for N…H (2.6%), (C) for H…N (2.6%), (D) for Au…Au (2.3%). In brackets, a given surface area included as a percentage of the total surface area is shown.

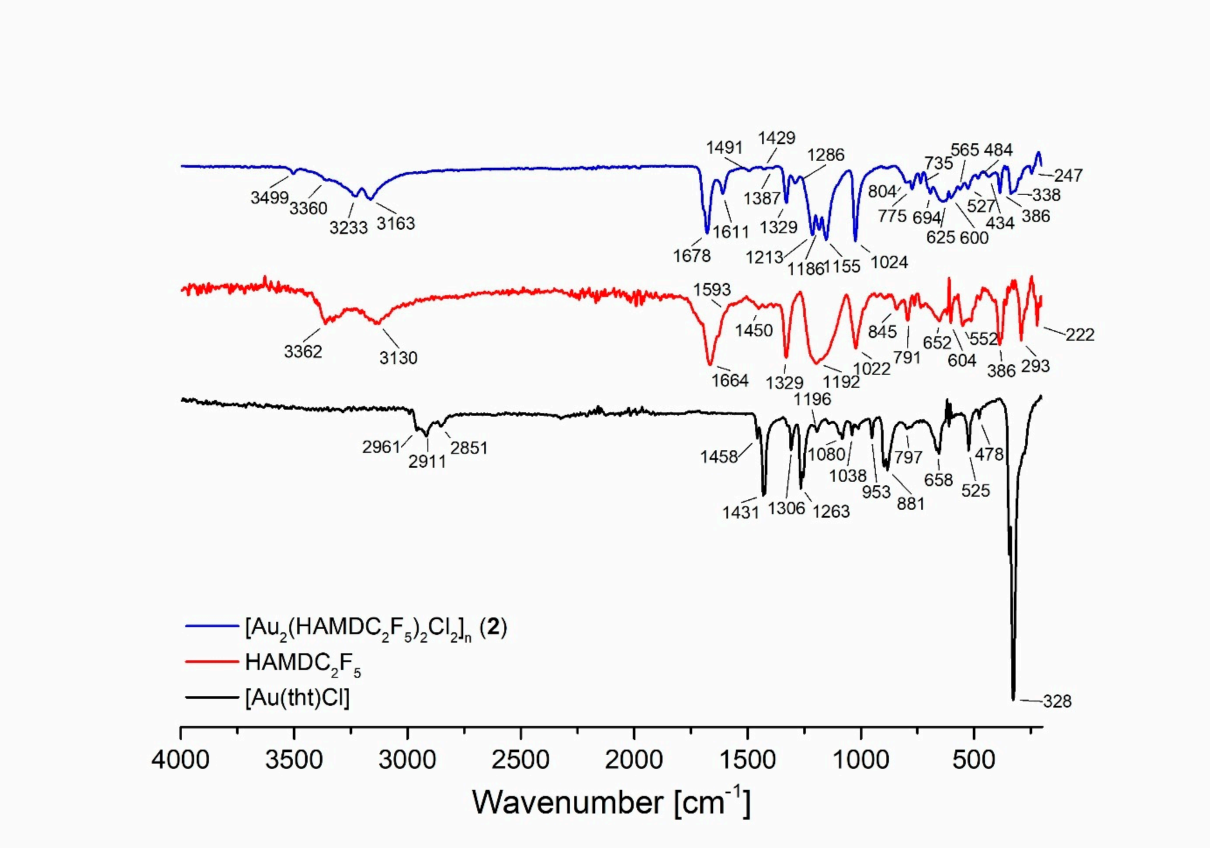

Figure S5. ATR-IR spectrum for the compound $[Au_2Cl_2(HAMDC_2F_5)_2]_n$ (**2**) (blue), $HAMDC_2F_5$ (red), and [Au(tht)Cl] (black).

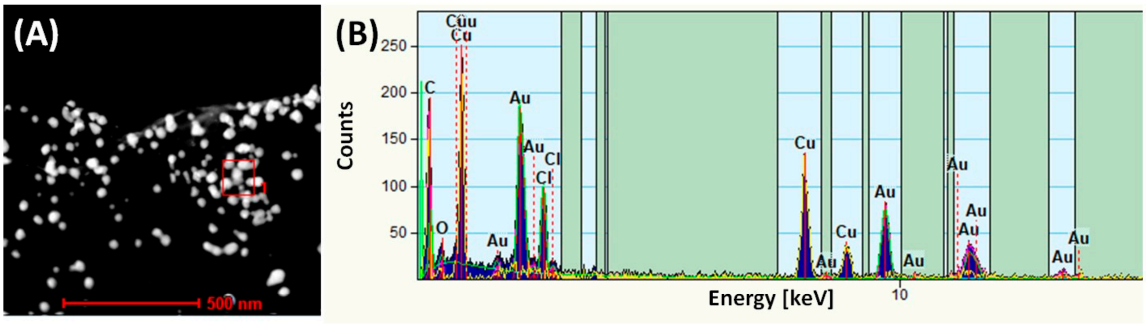

(C) Element	Atomic content of the element [at%]
Au	15.99
Cl	16.10
C*	63.61
O	4.28

*TEM grid covered with carbon

Figure S6. Results of TEM-EDX analysis obtained for the complex $[Au_2Cl_2(HAMDC_2F_5)_2]_n$ (**2**) after a few seconds of interaction with a focus high-energy electron beam (200 keV), (A) – TEM image, (B) TEM-EDX spectrum, and (C) atomic content of the element.

Table S3. Electron impact mass spectrometry (EI MS) results for the compound $[Au_4(\mu\text{-}AMDC_2F_5)_4]_n$ (**1**).

Fragments	m/z	Relative Intensity (RI) [%]				
		324 K	328 K	339 K	352 K	406 K
$[HN{=}C{=}N]^+$	41	5	–	3	2	–
$[HN{=}C{=}NH]^{+\bullet}$	42	47	2	4	4	1
$[HN{=}C{-}NH_2]^{+\bullet}$	43	7	3	6	6	10
$[FC_2H]^{+\bullet}$	44	25	25	52	55	100
$[FCN]^{+\bullet}$	45	69	1	8	12	2
$[CF_2]^+$	50	4	2	6	–	–
$[CF_3]^+$	69	60	12	40	38	59
$[CF_2CN]^+$	76	5	3	7	7	15
$[C_2F_4]^+$	100	6	3	4	4	7
$[C_2F_5]^+$	119	20	8	10	10	14
$[C_2F_4CN]^+$	126	4	2	4	4	11
$[NHCC_2F_5]^+$	146	1	2	1	–	1
$[NHNHC_2F_5]^+$	149	71	13	9	7	–
$[NHNH_2CC_2F_5]^{+\bullet}$	162	9	12	19	20	22
$[Au(NH)]^{+\bullet}$	212	16	–	–	–	–
$[Au(HNCNH)]^+$	239	2	4	4	5	1
$[Au(NHNH_2CCF)]^{+\bullet}$	271	–	1	–	–	–
$[Au(NHC_2F_5)]^{+\bullet}$	331	8	–	–	–	–
$[Au_2(NHNHCC_2F_5)_2]^{2+}$ /$[Au(NHNHCC_2F_5)]^{+\bullet}$	358	–	2	3	2	–
$[Au_2(NH_2)]^+$	410	–	1	1	–	–
$[Au_2(HNCNH)]^{2+}$	436	–	3	3	2	1
$[Au(NHNHCC_2F_5)_2]^+$	519	2	–	–	–	–
$[Au_2(NHNHCC_2F_5)]^+$	555	1	21	30	25	12
$[Au_2(NHNHCC_2F_5)(HCN)]^+$	582	–	6	6	5	1
$[Au_2(NHNHCC_2F_5)(HNCNH)]^+$	597	–	19	21	19	14
$[Au_2(NHNHCC_2F_5)_2]^{+\bullet}$	716	–	100	100	100	95
$[Au_3(NHNHCC_2F_5)_2]^+$	913	–	2	–	–	1
$[Au_4(NHNHCC_2F_5)]^+$	949	–	8	3	4	8

Table S4. Electron impact mass spectrometry (EI MS) results for the compound $[Au_2Cl_2(HAMDC_2F_5)_2]_n$ (**2**).

**Fragments**	**m/z**	**Relative Intensity (RI) [%]**			
		**336 K**	**339 K**	**344 K**	**363 K**
$[HCl]^{+\bullet}$	36	2	17	25	24
$[H_2{}^{35}Cl]^{+}$	37	9	67	100	100
$[H_2{}^{37}Cl]^{+}$	39	2	22	30	31
$[HN{=}C{=}NH]^{+\bullet}$	42	3	18	22	10
$[HN{=}C{-}NH_2]^{+\bullet}$	43	8	6	35	–
$[FC_2H]^{+\bullet}$	44	100	29	–	23
$[FCN]^{+\bullet}$	45	1	4	–	17
$[CF_2]^{+}$	50	2	1	7	3
$[CF_3]^{+}$	69	16	26	59	24
$[CF_2CN]^{+}$	76	–	6	17	5
$[C_4H_7S]^{+}$	87	–	100	8	10
$[CF_4]^{+\bullet}$ / $[C_4H_8S]^{+\bullet}$	88	1	62	6	9
$[C_2F_4]^{+}$	100	3	4	5	–
$[C_2F_5]^{+}$	119	12	18	29	10
$[C_2F_4CN]^{+}$	126	–	4	12	–
$[NHCC_2F_5]^{+}$	146	5	8	14	–
$[NHNH_2CC_2F_5]^{+\bullet}$	162	22	4	–	–
$[Au(NH)]^{+\bullet}$	212	–	11	52	52
$[Au(HNCNH_2)]^{+}$	240	–	3	–	–
$[Au(NHNH_2CCF)]^{+\bullet}$	271	–	2	4	3
$[Au(NHC_2F_5)]^{+\bullet}$	331	–	2	9	–
$[Au(NNCC_2F_5)]^{+\bullet}$	356	–	5	37	3
$[Au_2(NHNHCC_2F_5)_2]^{2+}$ /$[Au(NHNHCC_2F_5)]^{+\bullet}$	358	–	6	8	–
$[Au_2(NHNHCC_2F_5)_2]^{+\bullet}$	716	–	4	–	–
$[Au_3(NHNCC_2F_5)]^{+}$	751	–	3	–	–

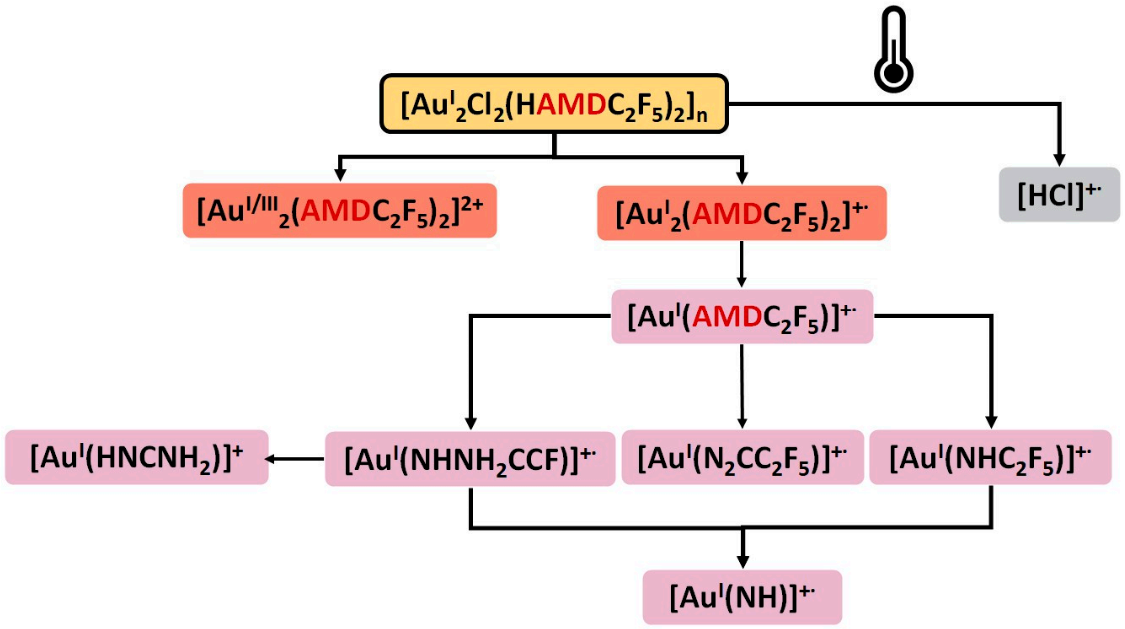

Figure S7. Fragmentation scheme for the most important ionsof [Au$_2$Cl$_2$(HAMDC$_2$F$_5$)$_2$]$_n$ (**2**) (HAMD – NHNH$_2$CC$_2$F$_5$; AMD – (NH)$_2$CC$_2$F$_5$)), assignment: grey – non-metalled ions, red – dinuclear ions, pink – mononuclear ions.

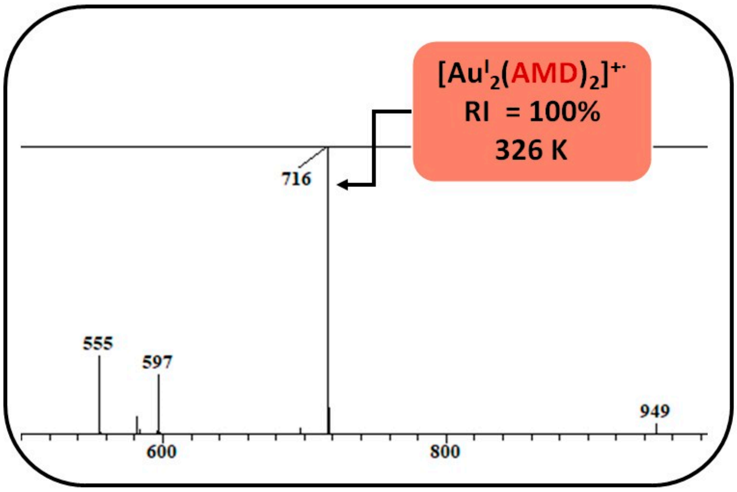

Figure S8. EI MS spectra, where the [Au$_2$(AMD)$_2$]$^{+\cdot}$ ion achieved relative intensity at the level of 100%.

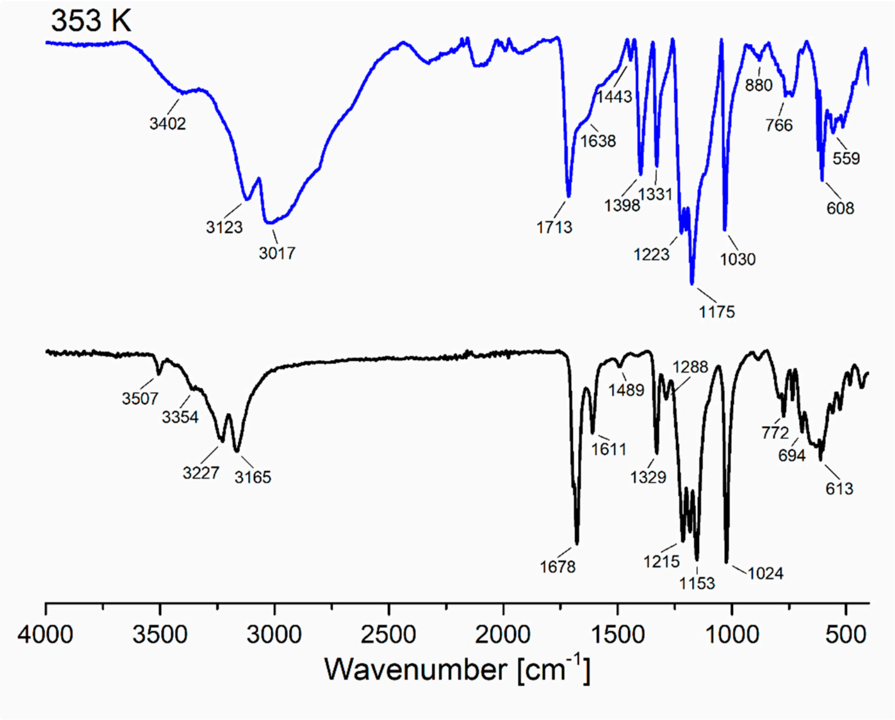

Figure S9. Infrared spectra for the compound $[Au_2Cl_2(HAMDC_2F_5)_2]_n$ (**2**) before (black) and after sublimation (blue) at 353 K (p = 10^{-2} mbar).

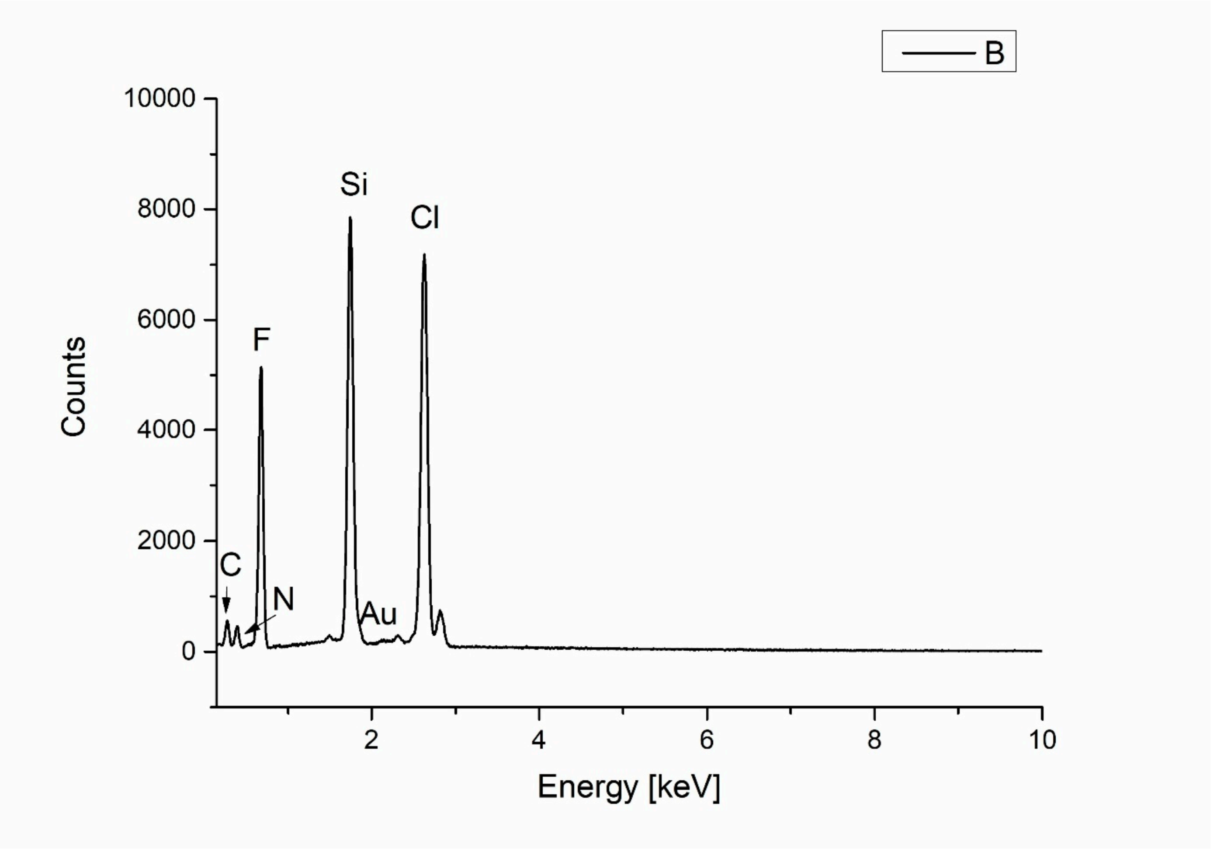

Figure S10. EDX spectra (20 keV) of product adsorbed on a Si(111) substrate during the sublimation test of $[Au_2Cl_2(HAMDC_2F_5)_2]_n$ (**2**).

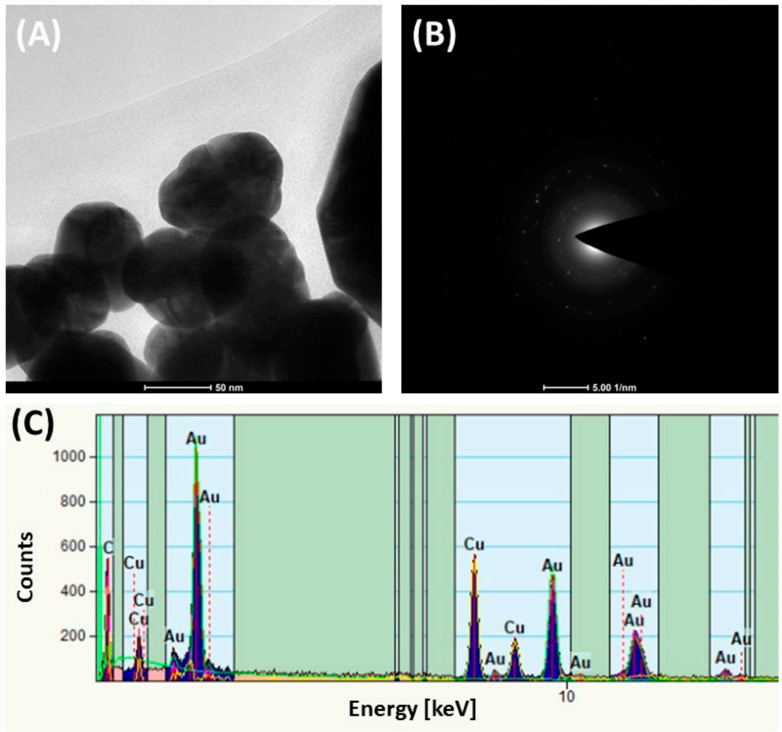

Figure S11. Transmission electron microscope image for gold nanoparticles formed after sublimation process for the complex $[Au_2Cl_2(HAMDC_2F_5)_2]_n$ (**2**) (A), TEM diffraction pattern (B), and energy-dispersive X-ray spectroscopy (EDX) spectrum (TEM grid covered with carbon) (C).

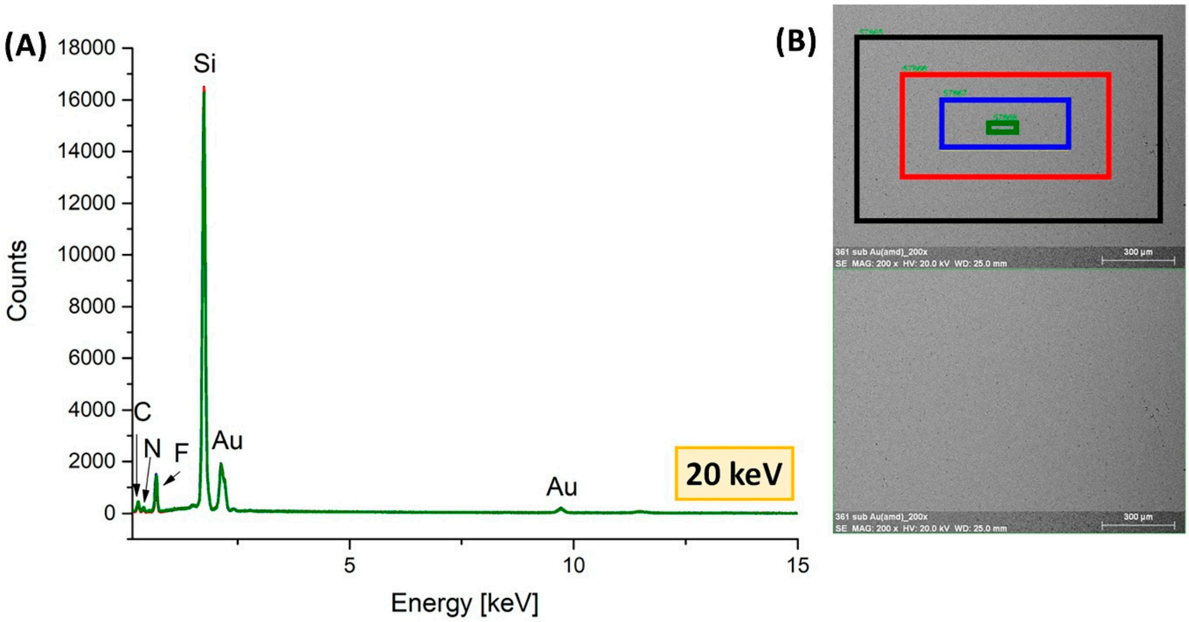

Figure S12. EDX spectra (20 keV) of sublimed $[Au_4(\mu\text{-}AMDC_2F_5)_4]_n$ (**1**) on a Si(111) substrate (Mag = 200×) (A). Top view SEM images of irradiated areas of the sublimed film (B): top: with marked areas corresponding to the EDX plots; bottom: morphological changes after irradiation; black, red, blue, and green rectangles – orderly with largest to smallest area of scanning, the colors correspond to changes in signals intensity in the EDX spectrum.

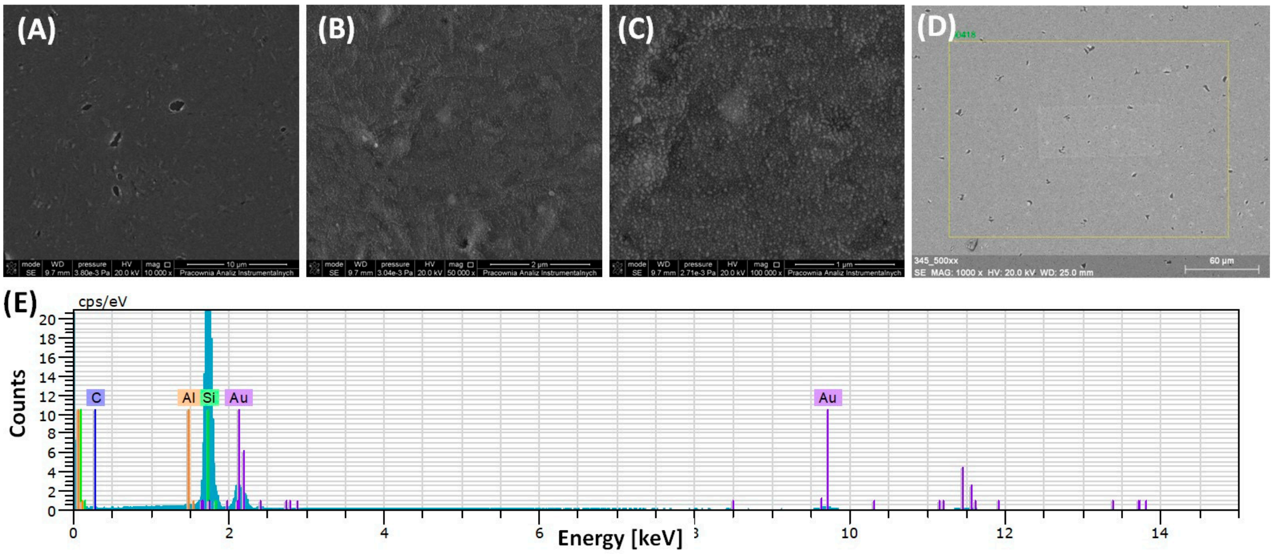

Figure S13. SEM images of vaporized layer of the compound $[Au_4(\mu\text{-}AMDC_2F_5)_4]_n$ (1) after heat treatment at 613 K: Mag = 10 000 x − (A); Mag = 50 000 x − (B); Mag = 100 000 x − (C). SEM image − (D) and EDX spectrum − (E) of studied area.
