# Supplementary material for: New Gold(I) Complexes as Potential Precursors for Gas-Assisted Methods: Structure, Volatility, Thermal Stability, and Electron Sensitivity
Source: Molecules. 2025 Jan 2;30(1):146. doi: 10.3390/molecules30010146 (PMC11721683; doi:10.3390/molecules30010146)

checkCIF/PLATON report

Structure factors have been supplied for datablock(s) nab26mo-3d

THIS REPORT IS FOR GUIDANCE ONLY. IF USED AS PART OF A REVIEW PROCEDURE FOR PUBLICATION, IT SHOULD NOT REPLACE THE EXPERTISE OF AN EXPERIENCED CRYSTALLOGRAPHIC REFEREE.

No syntax errors found. CIF dictionary Interpreting this report

Datablock: nab26mo-3d

Bond precision: C-C = 0.0040 A Wavelength=0.71073

Cell:	a=8.99189(19)	b=12.6776(3)	c=22.7927(5)
	alpha=90	beta=98.034(2)	gamma=90
Temperature:	100 K		

	Calculated	Reported
Volume	2572.77(10)	2572.77(10)
Space group	I 2/a	I 2/a
Hall group	-I 2ya	-I 2ya
Moiety formula	C12 H8 Au4 F20 N8	C12 Au4 F20 N8
Sum formula	C12 H8 Au4 F20 N8	C12 H8 Au4 F20 N8
Mr	1432.14	1432.13
Dx,g cm-3	3.697	3.697
Z	4	4
Mu (mm-1)	22.906	22.906
F000	2528.0	2528.0
F000'	2500.58	
h,k,lmax	11,15,28	11,15,28
Nref	2636	2625
Tmin,Tmax	0.346,0.632	0.277,0.854
Tmin'	0.031	

Correction method= # Reported T Limits: Tmin=0.277 Tmax=0.854
AbsCorr = GAUSSIAN

Data completeness= 0.996 Theta(max)= 26.370

R(reflections)= 0.0145(2368) wR2(reflections)= 0.0364(2625)

S = 1.035 Npar= 200

The following ALERTS were generated. Each ALERT has the format
test-name_ALERT_alert-type_alert-level.
Click on the hyperlinks for more details of the test.

Alert level C

```
PLAT042_ALERT_1_C Calc. and Reported MoietyFormula Strings  Differ     Please Check
              Calc: C12 H8 Au4 F20 N8
              Rep.: C12 Au4 F20 N8
PLAT911_ALERT_3_C Missing FCF Refl Between Thmin & STh/L=    0.600          7 Report
               -3  1  2,   4  0  4,   3  2  7,  -4  3  9,  -2  1 15,   3  8 19,
                5  5 20,
PLAT976_ALERT_2_C Check Calcd Resid. Dens.  1.08Ang From N12     .      -0.45 eA-3
PLAT976_ALERT_2_C Check Calcd Resid. Dens.  1.08Ang From N12     .      -0.45 eA-3
PLAT976_ALERT_2_C Check Calcd Resid. Dens.  0.92Ang From N12     .      -0.42 eA-3
```

Alert level G

```
FORMU01_ALERT_1_G  There is a discrepancy between the atom counts in the
            _chemical_formula_sum and _chemical_formula_moiety. This is
            usually due to the moiety formula being in the wrong format.
            Atom count from _chemical_formula_sum:   C12 H8 Au4 F20 N8
            Atom count from _chemical_formula_moiety:C12 Au4 F20 N8
PLAT007_ALERT_5_G Number of Unrefined Donor-H Atoms .............           4 Report
              H1    H2    H11   H12
PLAT764_ALERT_4_G Overcomplete CIF Bond List Detected (Rep/Expd) .       1.25 Ratio
PLAT883_ALERT_1_G No Info/Value for _atom_sites_solution_primary .     Please Do !
PLAT899_ALERT_4_G SHELXL2018 is Outdated   and Succeeded by SHELXL     2019/3 Note
PLAT910_ALERT_3_G Missing # of FCF Reflection(s) Below Theta(Min).          2 Note
                0  1  1,   0  0  2,
PLAT912_ALERT_4_G Missing # of FCF Reflections Above STh/L=  0.600          2 Note
PLAT933_ALERT_2_G Number of HKL-OMIT Records in Embedded .res File          9 Note
               -7  4 23,  -4  3  9,  -3  1  2,  -2  1 15,   3  2  7,   3  7 22,
                3  8 19,   4  0  4,   5  5 20,
PLAT969_ALERT_5_G The 'Henn et al.' R-Factor-gap value ..........      1.936 Note
              Predicted wR2: Based on SigI**2  1.88 or SHELX Weight  3.51
PLAT978_ALERT_2_G Number C-C Bonds with Positive Residual Density.          3 Info
```

   0 **ALERT level A** = Most likely a serious problem - resolve or explain
   0 **ALERT level B** = A potentially serious problem, consider carefully
   5 **ALERT level C** = Check. Ensure it is not caused by an omission or oversight
  10 **ALERT level G** = General information/check it is not something unexpected

   3 ALERT type 1 CIF construction/syntax error, inconsistent or missing data
   5 ALERT type 2 Indicator that the structure model may be wrong or deficient
   2 ALERT type 3 Indicator that the structure quality may be low
   3 ALERT type 4 Improvement, methodology, query or suggestion
   2 ALERT type 5 Informative message, check

It is advisable to attempt to resolve as many as possible of the alerts in all categories. Often the minor alerts point to easily fixed oversights, errors and omissions in your CIF or refinement strategy, so attention to these fine details can be worthwhile. In order to resolve some of the more serious problems it may be necessary to carry out additional measurements or structure refinements. However, the purpose of your study may justify the reported deviations and the more serious of these should normally be commented upon in the discussion or experimental section of a paper or in the "special_details" fields of the CIF. checkCIF was carefully designed to identify outliers and unusual parameters, but every test has its limitations and alerts that are not important in a particular case may appear. Conversely, the absence of alerts does not guarantee there are no aspects of the results needing attention. It is up to the individual to critically assess their own results and, if necessary, seek expert advice.

Publication of your CIF in IUCr journals

A basic structural check has been run on your CIF. These basic checks will be run on all CIFs submitted for publication in IUCr journals (*Acta Crystallographica*, *Journal of Applied Crystallography*, *Journal of Synchrotron Radiation*); however, if you intend to submit to *Acta Crystallographica Section C* or *E* or *IUCrData*, you should make sure that full publication checks are run on the final version of your CIF prior to submission.

Publication of your CIF in other journals

Please refer to the *Notes for Authors* of the relevant journal for any special instructions relating to CIF submission.

PLATON version of 22/08/2024; check.def file version of 21/08/2024

Datablock nab26mo-3d - ellipsoid plot

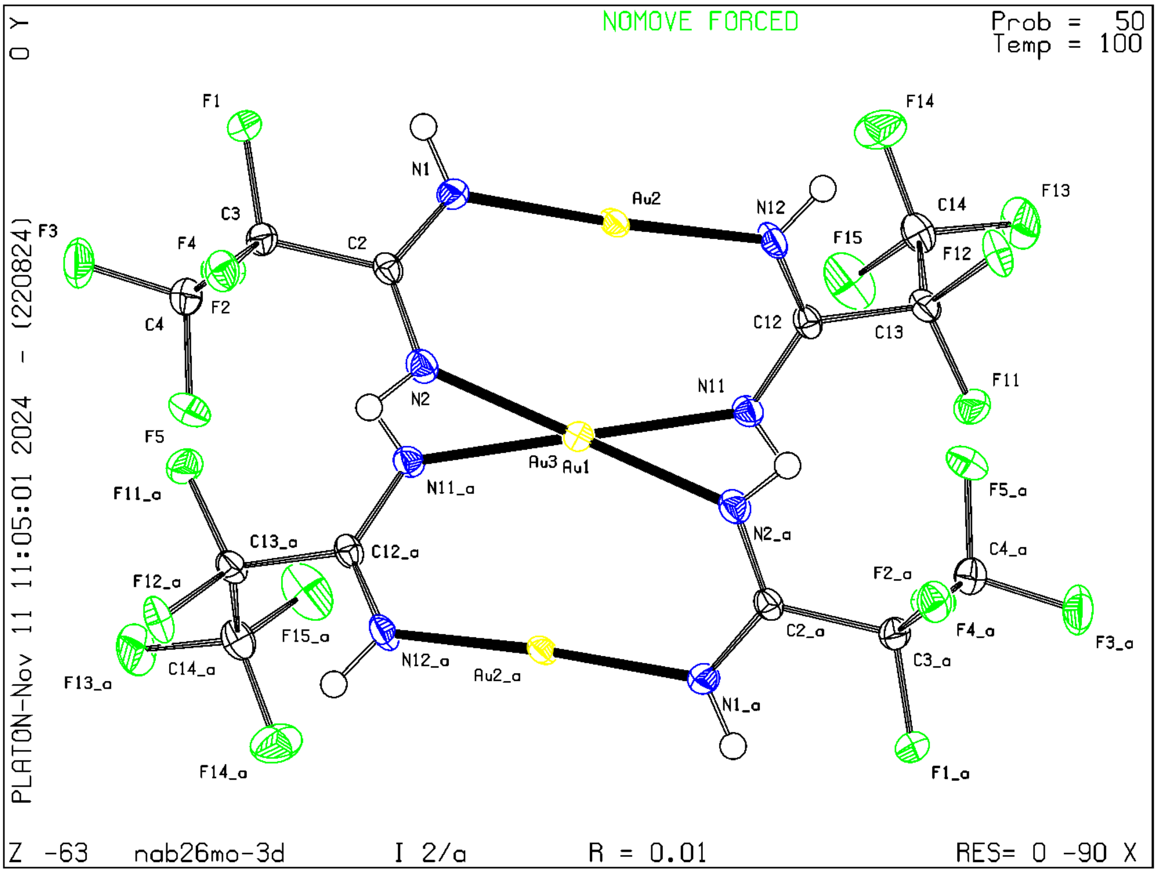

Supplement: Supplementary file 1 [file molecules-30-00146-s001.zip › nab26mo-3d-checkcif.pdf]
